# Enhanced IL-12 transgene expression improves oncolytic viroimmunotherapy

**DOI:** 10.3389/fimmu.2024.1375413

**Published:** 2024-06-04

**Authors:** Yeaseul Kim, Uksha Saini, Doyeon Kim, Ilse Hernandez-Aguirre, Jack Hedberg, Alexia Martin, Xiaokui Mo, Timothy P. Cripe, James Markert, Kevin A. Cassady, Ravi Dhital

**Affiliations:** ^1^ Center for Childhood Cancer Research, Abigail Wexner Research Institute at Nationwide Children’s Hospital, Columbus, OH, United States; ^2^ Department of Biomedical Informatics, The Ohio State University College of Medicine, Columbus, OH, United States; ^3^ College of Medicine, The Ohio State University, Columbus, OH, United States; ^4^ Department of Pediatrics, The Ohio State University College of Medicine, Columbus, OH, United States; ^5^ Department of Neurosurgery, The University of Alabama at Birmingham, Birmingham, AL, United States; ^6^ Department of Pediatrics, Division of Pediatric Infectious Diseases, Nationwide Children’s Hospital, Columbus, OH, United States

**Keywords:** IL-12, oncolytic herpes simplex virus (oHSV), malignant peripheral nerve sheath tumor (MPNST), CD4+ T cells, dendritic cells, Interferon-gamma, oncolytic virotherapy (OV), MHC-II upregulation

## Abstract

**Introduction:**

Malignant peripheral nerve sheath tumors (MPNSTs) are aggressive sarcomas with unacceptably low cure rates occurring often in patients with neurofibromatosis 1 defects. To investigate oncolytic Herpes Simplex Virus (oHSV) as an immunotherapeutic approach, we compared viral replication, functional activity, and immune response between unarmed and interleukin 12 (IL-12)-armed oncolytic viruses in virus-permissive (B109) and -resistant (67C-4) murine MPNSTs.

**Methods:**

This study compared two attenuated IL-12-oHSVs with γ134.5 gene deletions (Δγ134.5) and the same transgene expression cassette. The primary difference in the IL-12-oHSVs was in their ability to counter the translational arrest response in infected cells. Unlike M002 (Δγ134.5, mIL-12), C002 (Δγ134.5, mIL-12, IRS1) expresses an HCMV IRS1 gene and evades dsRNA activated translational arrest in infected cells.

**Results and discussion:**

Our results show that oHSV replication and gene expression results in vitro were not predictive of oHSV direct oncolytic activity in vivo. Tumors that supported viral replication in cell culture studies resisted viral replication by both oHSVs and restricted M002 transgene expression in vivo. Furthermore, two IL-12-oHSVs with equivalent transcriptional activity differed in IL-12 protein production in vivo, and the differences in IL-12 protein levels were reflected in immune infiltrate activity changes as well as tumor growth suppression differences between the IL-12-oHSVs. C002-treated tumors exhibited sustained IL-12 production with improved dendritic cells, monocyte-macrophage activity (MHCII, CD80/CD86 upregulation) and a polyfunctional Th1-cell response in the tumor infiltrates.

**Conclusion:**

These results suggest that transgene protein production differences between oHSVs in vivo, in addition to replication differences, can impact OV-therapeutic activity.

## Introduction

Malignant Peripheral Nerve Sheath Tumors (MPNSTs) are rare, aggressive soft tissue sarcomas, which most often occur as a neoplasm in patients with neurofibromatosis 1 (NF1). MPNSTs exhibit resistance to non-surgical treatment due to their high metastatic potential and rates of relapse, low response rates to chemotherapy, and inclination for fast disease progression (1, 2). Prognosis for MPNSTs is poor, with a pooled recurrence rate of 42%, distant metastasis rate of 27%, and mortality rate of 26% (3). Moreover, overall mortality of MPNSTs have been found to remain largely unaffected by degree of tumor resection or use of radiation therapy as an adjuvant therapy (3). As such, there remains a critical need to continue efforts towards developing effective therapies for MPNSTs. Oncolytic virotherapy is a novel therapy alternative to chemo- or radiation therapy used to treat MPNSTs (4). Oncolytic viruses are modified viruses that selectively replicate in malignant tumors to induce direct cytotoxic or immune-related anti-tumor activity and are often equipped to express transgenes that can heighten their therapeutic effect (5). Oncolytic Herpes Simplex Virus (oHSV) is one such oncolytic virus that has been proposed as a promising cancer therapeutic, with ongoing progress in clinical trials and one agent already approved by the FDA for the treatment of melanoma (6–9).

In the present study, we tested the anti-tumor effects of two different murine (m)IL-12-expressing oHSVs in virus-permissive (B109) and -resistant (67C-4) murine MPNSTs. Both oHSVs contain deletions in the principal neurovirulence gene, γ_1_34.5, that render it safe to use in patients but maintains selective replication in tumor cells (10). One of the Δγ_1_34.5 IL-12 oHSVs, M002, has previously been demonstrated to enhance survival of mice bearing intracranial brain tumors and lack significant systemic or neurologic toxicity following treatment in a nonhuman primate model (11, 12). M032, an oHSV with the exact configuration as M002, but expresses human (h)IL-12 in lieu of mouse IL-12, has completed Phase I clinical trial (Clinical Trial NCT02062827). The other IL-12 oHSV tested, C002, is derived from C134, a Δγ_1_34.5 oHSV that expresses the HCMV gene IRS1. Addition of the HCMV gene enables the attenuated oHSV to evade the infected cell’s dsRNA-activated translational arrest response enhancing oHSV replication in tumor cells without restoring wild type neurovirulence and thus improving the therapeutic response (13, 14).

IL-12 is a heterodimeric cytokine, primarily produced by antigen presenting cells, that induces IFN-γ production, promotes NK-cell mediated cytotoxicity, and enhances T cell responses (15). It also facilitates the differentiation of naïve CD4(+) T cells to T-helper 1 cells and promotes the differentiation of naïve B cells into IgM-secreting plasma cells (16, 17). The capacity of IL-12 to regulate both the innate and adaptive arms of the immune response, coupled with its intrinsic anti-tumor effects, has rendered it an attractive immunotherapy over the last several decades (18, 19).

We hypothesized that IL-12 transgene expression by an oHSV with improved viral gene expression would enhance the oHSV efficacy. We compared the functional effects of the viral gene expression and replication of a Δγ_1_34.5 IL-12 expressing oHSV (M002) and Δγ_1_34.5, IRS1 IL-12-expressing oHSV (C002) in the treatment of murine MPNSTs, B109 and 67C-4. This study highlights nuanced differences between oHSVs in biologically active IL-12 production despite similar replication within murine sarcomas. The variability in IL-12 production associates with distinct cytokine-related effects within the tumor immune microenvironment. oHSVs generating elevated IL-12 demonstrated superior therapeutic efficacy across both tumor models. These findings underscore the critical role of viral IL-12 expression in initiating antitumoral immune response and enhancing the efficacy of oncolytic virotherapy.

## Materials and methods

### Cell lines and viruses

67C-4 was kindly provided by Dr. Nancy Ratner (University of Cincinnati, Cincinnati, OH), and B109 by Dr. Steven Carroll (University of South Carolina, Charleston, SC). Both MPNST cell lines were maintained in DMEM, supplemented with 10% FBS. Vero cells were maintained in DMEM with 5% BGS and were obtained from ATCC (Manassas, VA). Viruses HSV-1(F), R3616, C101, and C134 have been described previously (10, 13, 20). In brief, HSV-1(F) is an F strain wild type HSV, and all the recombinants used in this study were F strain derived. R3616, the Δγ_1_34.5 recombinant, was graciously provided by Dr. Bernard Roizman (University of Chicago, Chicago, IL). C101 is a Δγ_1_34.5 virus derived from R3616 that expresses EGFP (13), and C134 is a Δγ_1_34.5 virus derived from C101 that contains the HCMV IRS1 gene under control of the HCMV IE promoter in the UL3/UL4 intergenic region (13). M002 is a Δγ_1_34.5 virus that expresses mIL-12 and was kindly provided by Dr. James Markert (University of Alabama at Birmingham, Birmingham, AL). C002 is a C134-derived virus that contains the same mIL-12 bicistronic cassette as M002 in the γ_1_34.5 locus and expresses mIL-12.

### Viral recovery plaque assay and qPCR

67C-4 and B109 cells were plated into clear, 24-well flat-bottom polystyrene tissue culture plates and allowed to adhere overnight at 37°C. The following day, cells were infected with C101, C134, M002, or C002 at a multiplicity of infection of one (MOI=1) for 2 hours with virus diluted in 225µL infection medium (DMEM, 1% BGS). The medium was replaced with growth medium (DMEM, 10% FBS) after the 2 hours of incubation. At 24- and 48-hours post infection, cells were scraped off wells, lysed, centrifuged to pellet cell debris, supernatants were serially diluted in infection medium (DMEM, 1% BGS), and virus recovery was measured by limiting dilution plaque assay using Vero monolayers. Viral plaques were quantified 2 days post-infection from May-Grünwald/methanol-stained plaque dishes.

### Viral load qRT-PCR

Flank tumors were harvested and homogenized by mechanical disruption using scissors and the TissueRuptor II (Qiagen). DNA was extracted from aliquots of tissue homogenates using the QIAamp DNA Mini Kit (Qiagen). 200ng gDNA, forward and reverse primers at a final concentration of 300nM, probe at a final concentration of 100nM, and Taqman Gene Expression Mastermix (Applied Biosystems) were mixed and aliquoted into a qPCR plate. Following primers and probe used were used: HSV Pol F: 5’-ACCGCCGAACTGAGCAGAC-3’, HSV Pol R: 5’-TGAGCTTGTAATACACCGTCAGGT-3’, HSV Pol Probe: 5’-CGCGTACACCAACAAGCGCCTG-3’ (14). Amplification protocol included 2 min Hold at 50°C, 10 min Hold at 95°C, 35x Cycle of 15 sec at 95°C and 1 min at 60°C. Samples were quantified using a standard curve generated using a log diluted (+) control standard cosmid DNA and normalized to the amount of starting DNA.

### IL-12 qRT-PCR

Flank tumors were harvested and homogenized by mechanical disruption using scissors and the TissueRuptor II (Qiagen). Aliquots of tissue homogenate were stored in RNAlater at -80°C until the day of assay. RNA was extracted from thawed samples using *Quick*-RNA Miniprep Plus Kit (Zymo Research) and cDNA produced from equivalent mass of RNA from each sample using the RevertAid RT Kit (Thermo Scientific) followed by qPCR using SYBR Green 2X Mastermix (Applied Biosystems). Amplification protocol included 2 min UDG Activation at 50°C, 10 min Dual-Lock DNA Polymerization at 95°C, and a 40x Cycle of 15 sec at 95°C and 30 sec at 62°C. Specificity of reaction was verified with a melt curve analysis, and samples quantified using a standard curve generated using a log diluted mIL12 (+) control standard plasmid. Primers used (500nM final concentration) are as follows: mIL-12 P35 Sybr F: 5’-GAAACATTATTCCTGCACTGCTGA-3’, mIL-12 p35 875 Sybr R: 5’-GCAACTCTCGTTCTTGTGTAGTTC-3’. IL-12 transcript quantity was normalized to the amount of starting RNA.

### Animal tumor studies

Animal studies were approved by the Nationwide Children’s Hospital Institutional Animal Care and Use Committee (IACUC, protocol number AR19–00177) and performed in accordance with guidelines established by the Department of Defense and the National Institutes of Health (NIH) Guide for the Care and Use of Laboratory Animals. Two syngeneic C57BL/6 tumor models, a flank 67C-4 MPNST model and flank B109 MPNST model, were used in these studies. For 67C-4 flank tumor studies, 3- to 4-week-old C57BL/6 mice were obtained from Envigo (Frederick, MD) and implanted subcutaneously with 4×10^6^ cells in 50µL of phosphate buffered saline (PBS)/flank. B109 flank tumor studies were conducted with 3- to 4-week-old C57BL/6 mice from Charles River (Wilmington, MA) implanted subcutaneously with 6 x10^6^ to 8×10^6^ cells in 50µL of phosphate buffered saline (PBS)/flank in independent studies. Tumor sizes in both studies were measured biweekly by caliper after implantation, and tumor volume was calculated. Due to differences in tumor growth rate and tumor establishment rates, tumor size at the time of treatment differed between the 67C-4 and B109 tumors. Animals were randomized into treatment groups when tumors reached 60–200mm^3^ (67C-4) or 64–600mm^3^ (B109). Tumors were then treated with saline or oHSV C134, M002, or C002 (3×10^7^ PFU in 50µL PBS)/flank intratumorally (ITu). Tumor measurement was terminated upon reaching the IACUC endpoint criteria of 3000mm3 volume per mouse, at which point the mice were euthanized. Flank tumor growth studies were repeated to ensure biological reproducibility. Samples sizes for the studies were as follows: 67C-4 tumor growth study: PBS (n=6), C134 (n=6), M002 (n=7), C002 (n=7). B109 tumor growth study: PBS (n=5), C134 (n=4), M002 (n=8), C002 (n=5). Immune phenotype study: n=8/group.

### Spectral flow cytometry

Flank tumors were harvested and homogenized by mechanical disruption using scissors and the TissueRuptor II (Qiagen). Tumor lysates were then passed through a 70µm cell strainer, pelleted, and leukocytes were separated by Ficoll overlay (Ficoll-Paque PREMIUM 1.084). Brefeldin A solution (1000X, Biolegend, CA) was added to cells at a final concentration of 1X and incubated at 37°C for 3 hours. Following this incubation, dead cells were stained with Zombie NIR Live Dead Stain (Biolegend, CA) for 20 minutes at 37°C. Samples were washed with FACS buffer (PBS, 5% FBS, 0.1% NaN_3_) and stained for surface markers for 40 minutes at room temperature. Samples were washed again and fixed with FoxP3 Fixation/Permeabilization working solution (eBioscience, San Diego, CA) for 30 minutes at room temperature. Fixed samples were then washed with 1X Permeabilization buffer and stained overnight at 4°C for intracellular targets. Samples were washed a final time, resuspended in FACS buffer, and acquired via Cytek Aurora spectral flow cytometer outfitted with five lasers (355nm, 405nm, 488nm, 561nm and 640nm) using SpectralFlo version 3.1.0 (Cytek Biosciences, Fremont, CA). Data was analyzed using FlowJo v10.8.1 (BD Biosciences). Markers of interest were expressed as a percentage of the parent cell population, total live CD45(+) cells, or absolute event counts. The following antibodies were used: a) Surface: BUV395-CD103, StarBright UV445-CD25, BUV496-NK1.1, BUV563-F4/80, BUV661-CD86, BUV737-CD49b, eFluor 450-Ly6C, BV570-CD11b, BV650-CD80, NovaFluor Blue 510-MHC-II, NovaFluor Blue 585-CD62L, NovaFluor Blue 61–70S-CD170, PerCP-CD45, PerCP-eflour 710-TCRγδ, RB780-CD19, Spark YG 593-CD44, PE-Fire 640-CD11c, PE-Cy5-CD69, NovaFluor Yellow 690-CD4, PE/Fire 700-CD206, PE-Fire 810-CD3, APC-H7- CD8 and APC-Fire 810-Ly6G b) Intracellular/nuclear: BV421-Granzyme B, SuperBright 436- IL-21, BV510-IL-17A, BV711-IL-4, BV750-TNF-α, PE-efluor 610-IL-10, APC-IFN-γ and Alexa Fluor 647-FoxP3. The working dilution of each antibody was initially validated by comparing with unstained controls, isotype controls, and fluorescence-minus-one staining using splenocytes from non-tumor bearing mice. All antibodies were purchased from commercial vendors: BD Biosciences (Franklin Lakes, NJ), Biolegend (San Diego, CA), ThermoFisher (Waltham, MA), R & D Systems (Minneapolis, MN), and Bio-Rad laboratories (Hercules, CA).

### Enzyme-linked immunosorbent assay and multi-parameter analyte immunoassay (ProcartaPlex)

For *in vitro* studies, cells were infected with M002 and C002 at MOI=1. Culture supernatants were collected at 48 hours to quantify secreted IL-12 cytokine using the mouse IL-12 (p70) ELISA MAX Deluxe Set (BioLegend, CA) following the manufacturer’s protocol. For *in vivo* studies, flank tumors were harvested and homogenized on ice by mechanical disruption using scissors and a TissueRuptor II (Qiagen). Aliquots of tissue homogenate were then stored at -80°C until the day of assay. Samples were prepared by thawing and incubating with an equal volume of SDS-free RIPA lysis buffer (diH_2_O, 150mM Sodium Chloride, 50mM Tris-HCL, 1% Nonidet P-40, 0.5% Sodium deoxycholate) for 30 minutes at room temperature. The lysed samples were centrifuged, supernatants were collected and analyzed with the ELISA or cytokine bead array (ProcartaPlex, ThermoFisher, Waltham, MA) according to the manufacturer’s protocol. For the multiplex assay, analytes measured include IL-12 (p40), IFN-γ, and TNF-α. Samples were acquired using Bio-Plex 200 (Bio-Rad, Hercules, CA). Cytokines values were normalized for volumes of media suspended during mechanical disruption and depicted as picograms (pg) per ml (pg/ml) or pg/1000mm^3^ volume of tumor.

### Statistical analysis

For experiments with two groups, Mann-Whitney 2-sample tests were conducted. In experiments involving four groups, the Kruskal-Wallis test was initially applied, followed by pairwise comparisons using Dunn’s method. For viral load RT-qPCR experiments, technical replicates were first averaged, transformed by log2, and analyzed with ANOVA. Longitudinal tumor growth measures were log2 transformed to meet normality assumptions. To account for observational dependencies across days for each tumor, mixed effect modeling was performed on the transformed data, followed by group comparisons at specific time points. TGR (tumor growth rates) were estimated and compared using mixed effect model in SAS (proc mixed). To control type I error, Hochberg method was employed for adjusting multiplicities related to primary hypotheses. Adjusted p-value<0.05 was considered significant. Dunn’s tests were performed using Minitab 21 (Minitab LLC; State College, PA), and other statistical analyses were carried out using SAS 9.4 (SAS Institute; Kary, NC) or Prism GraphPad v10.1.0 (GraphPad Software Inc; San Diego, CA). Statistical significance is designated with asterisks as follows: *p<0.05, **p<0.01, ***p<0.001, ****p<0.0001.

## Results

### oHSV activity (replication and transgene expression) differed in B109 and 67C-4 *in vitro*

**Figure 1:**
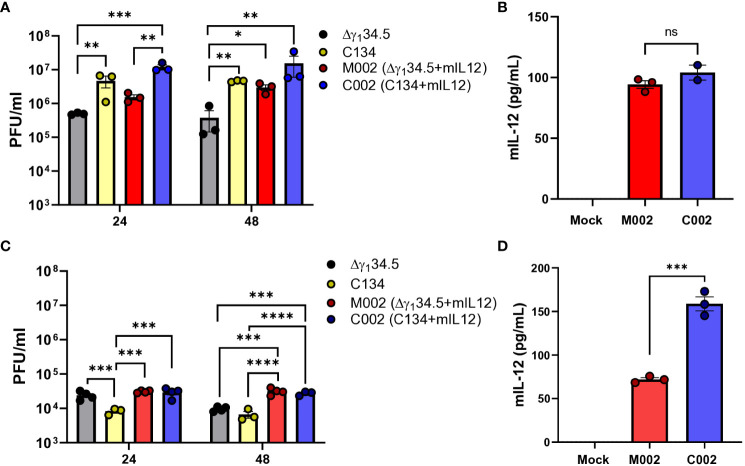
Viral recovery and IL-12 production levels of M002 and C002 in B109 and 67C-4 MPNST cells *in-vitro*. B109 or 67C-4 MPNST cells were plated into polystyrene tissue culture plates and allowed to adhere overnight at 37°C. Cells were infected the following day with C101, C134, M002, and C002 at a multiplicity of infection (MOI) of 1. At 24- and/or 48-hours post infection, cells were harvested, and virus recovery was measured by limiting dilution plaque assay. Culture supernatants were quantified for IL-12 cytokine protein using mouse IL-12 (p70) ELISA MAX Deluxe Set following the manufacturer’s protocol. **(A)** Viral recovery of Δγ_1_34.5 oHSV, C134, M002, and C002 from B109 cells *in vitro* were similar at 24 hours post infection, but viral recovery of C002 was greater than Δγ_1_34.5 oHSV at 48 hours post infection. **(B)** M002 and C002 produced similar levels of IL-12 protein in B109 cells *in vitro*. **(C)** Viral recovery of M002 and C002 from 67C-4 cells *in vitro* was greater than that of C134 at 24 hours post infection, while M002 and C002 viral recovery was greater than that of both Δγ_1_34.5 oHSV and C134 at 48 hours post infection. **(D)** C002 produced greater levels of IL-12 protein than did M002 in 67C-4 cells *in vitro*. *p<0.05, **p<0.01, ***p<0.001, ****p<0.0001. ns, not significant.

To investigate the replication and gene expression of IL-12 expressing oncolytic viruses (IL-12 oHSVs), we examined two IL-12 oHSVs, M002 (Δγ_1_34.5, mIL-12) and C002 (Δγ_1_34.5, IRS1, mIL-12), in two representative murine MPNST lines (B109 and 67C-4) based upon our previous studies highlighting oHSV replication and gene expression differences in these MPNST lines (7). Cells were seeded and infected at MOI=1 with either control oHSV (Δγ_1_34.5 or C134) or IL-12 oHSV (M002 or C002) for 24 and 48 hours. Cells were harvested, lysed, and assessed for viral replication by plaque assay. Culture supernatants were quantified for secreted Interleukin-12 (IL-12) cytokine using ELISA. The B109 MPNST tumor line supported oHSV replication *in vitro* (**Figure 1A**). oHSV recovery ranged from 4x10^5^ - 1.5x10^7^ PFU/ml. At 24 hours post-infection, C002 (Δγ_1_34.5, IRS1, mIL-12) produced significantly greater infectious virus than did M002 (1.19x10^7^ PFU/ml vs. 1.5x10^6^ PFU/ml, *p=0.008*) or the control Δγ_1_34.5 oHSV (1.19x10^7^ PFU/ml vs. 4.9x10^5^ PFU/ml, *p=0.0064*). Similarly, C134 generated significantly greater infectious virus than the control Δγ_1_34.5 oHSV (4.66x10^6^ PFU/ml vs. 4.9x10^5^ PFU/ml, *p=0.0089*). By 48 hours, virus recovery was comparable between M002 and C002 (*p>0.05*) while replication of the control Δγ_1_34.5 oHSV lagged (1.54x10^7^ PFU/ml vs. 3.7x10^5^ PFU/ml*, p=0.0017*). Similar to the viral recovery studies, both M002 and C002 produced equivalent IL-12 (p70) after B109 infection (p>0.05, **Figure 1B**).

In contrast, the 67C-4 MPNST cell line restricted efficient oHSV replication (21). Viral recovery studies showed over 500x less virus recovered when compared to B109 MPNST cells (**Figures 1A, C**). While M002 and C002 demonstrated similar replication kinetics and viral recovery in 67C-4 cells at 24h (3.07x10^4^ PFU/ml vs. 2.92x10^4^ PFU/ml, *p>0.05*) and 48h (3.14x10^4^ PFU/ml vs. 2.75x10^4^ PFU/ml, *p>0.05*) post-infection (**Figure 1C**), they both exhibited statistically higher recovery compared to C134 control virus at both time points. At 48 hours, both M002 and C002 also surpassed Δγ_1_34.5 oHSV in viral recovery. These results indicate superior replication of both IL-12 viruses compared to the non-IL-12 control viruses. However, it is essential to note that the observed statistical differences in viral recovery between the IL-12 and non-IL-12 oHSVs may not necessarily translate into significant biological implications, as they fall within the titering variability of ½ log. Notably, IL-12 production in 67C-4 cells differed between M002 and C002 (**Figure 1D**), with C002 demonstrating a 2.2-fold increase in IL-12 levels compared to M002 (158.73 pg/ml vs 71.9 pg/ml, *p=0.0003*). In summary, while the IL-12 viruses exhibited similar replication dynamics, the C002 demonstrated enhanced IL-12 protein production in the more restrictive 67C-4 MPNST line.

### C002 produced sustained IL-12 and suppressed B109 MPNST tumor growth *in vivo*

**Figure 2:**
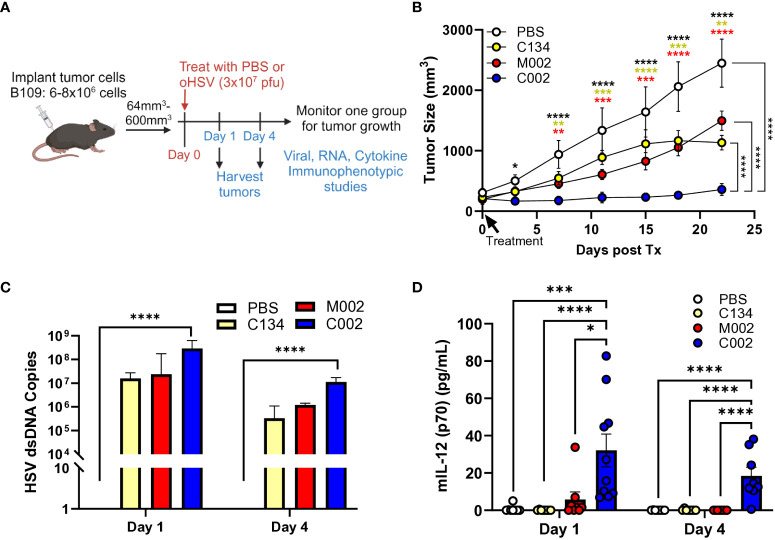
C57BL/6-based B109 MPNST summary. Three- to 4-week-old C57BL/6 mice were implanted subcutaneously with 6–8×10^6^ cells in 50µL of phosphate buffered saline (PBS)/flank. When tumor sizes reached 64–600mm^3^, animals were randomized into treatment groups by tumor size to ensure a similar average tumor size per cohort. Tumors were then treated with saline or oHSV C134, M002, or C002 (3×10^7^ PFU in 50µL PBS)/flank intratumorally (ITu). The tumors were measured twice/week until endpoint for tumor growth studies. A separate cohort of mice were sacrificed at 1- and 4- days post-treatment, tumors harvested and homogenized by mechanical disruption. DNA was extracted from aliquots of tissue homogenates and qPCR-amplified for viral copies. Another aliquot of tissue homogenates were incubated with an equal volume of SDS-free RIPA lysis buffer, centrifuged, supernatants collected, and analyzed with mouse IL-12 (p70) ELISA MAX Deluxe Set **(A)** Schematic of experimental design of B109 animal tumor studies. **(B)** A single intratumoral dose of C002 suppressed B109 flank tumor growth significantly than did a single dose of saline (p<0.01), C134 (p<0.01), or M002 (p<0.01). PBS (n=5), C134 (n=4), M002 (n=8), C002 (n=5). **(C)** All oHSV treatments produced similar levels of vDNA at both 1- and 4-days post treatment, and only C002 treatment produced statistically greater vDNA levels than PBS treatment (p<0.0001). **(D)** C002 produced greater levels of IL-12 (p70) protein than all other virus cohorts produced at both 1- and 4-days post treatment in B109 flank tumors detected by ELISA. Day 1: PBS (n=8), C134 (n=8), M002 (n=8), C002 (n=10). Day 4: PBS (n=8), C134 (n=8), M002 (n=8), C002 (n=8). *p<0.05, **p<0.01, ***p<0.001, ****p<0.0001.

To evaluate the therapeutic potential of oHSVs in tumors that support virus replication, we utilized B109 murine MPNSTs, known for their support of oHSV replication *in vitro*, and performed tumor growth studies in B109 flank tumors. We hypothesized that M002 and C002 exert similar therapeutic effects based on our *in-vitro* results; however, our results showed significant differences in anti-tumor activity between the two IL-12 oHSVs. We established B109 tumors in immune competent C57BL/6 mice, randomized, and treated tumors with a single dose of saline or oHSV (3x10^7^ PFU) intratumorally (**Figure 2A**). Contrary to our expectations, neither M002 nor C134 provided therapeutic benefit when compared to saline control treatment in this murine MPNST tumor model (**Figure 2B**). In contrast, compared to saline, C134, and M002 treatments, a single dose of C002 suppressed tumor growth *(p<0.0001)* and improved survival in treated mice (**Supplementary Figure 1A**).

Next, we examined HSV DNA levels in treated tumors at Day 1 (D1) and Day 4 (D4) post-treatment by TaqMan qPCR assay. All oHSVs generated nearly identical levels of viral DNA even at D4 following treatment, although at reduced quantities compared to D1 (**Figure 2C**).

Next, we investigated differences in IL-12 protein production between virus-treated tumors by measuring IL-12 (p70) levels using ELISA (BioLegend) at D1 and D4 post-treatment from clarified tumor tissue homogenates. C002 treatment consistently generated higher IL-12 protein levels compared to both M002 and C134 at both timepoints (**Figure 2D**). Notably, no significant differences in the IL-12 levels were found between D1 and D4 in C002-treated tumors. In summary, only C002 treatment produced measurable IL-12 levels above background, persisting up to 4 days post-treatment. Conversely, M002 did not produce detectable IL-12 protein above background levels despite equivalent viral recovery. These results underscore the distinct *in vivo* therapeutic efficacy of C002 in the B109 murine MPNST model.

**Figure 3:**
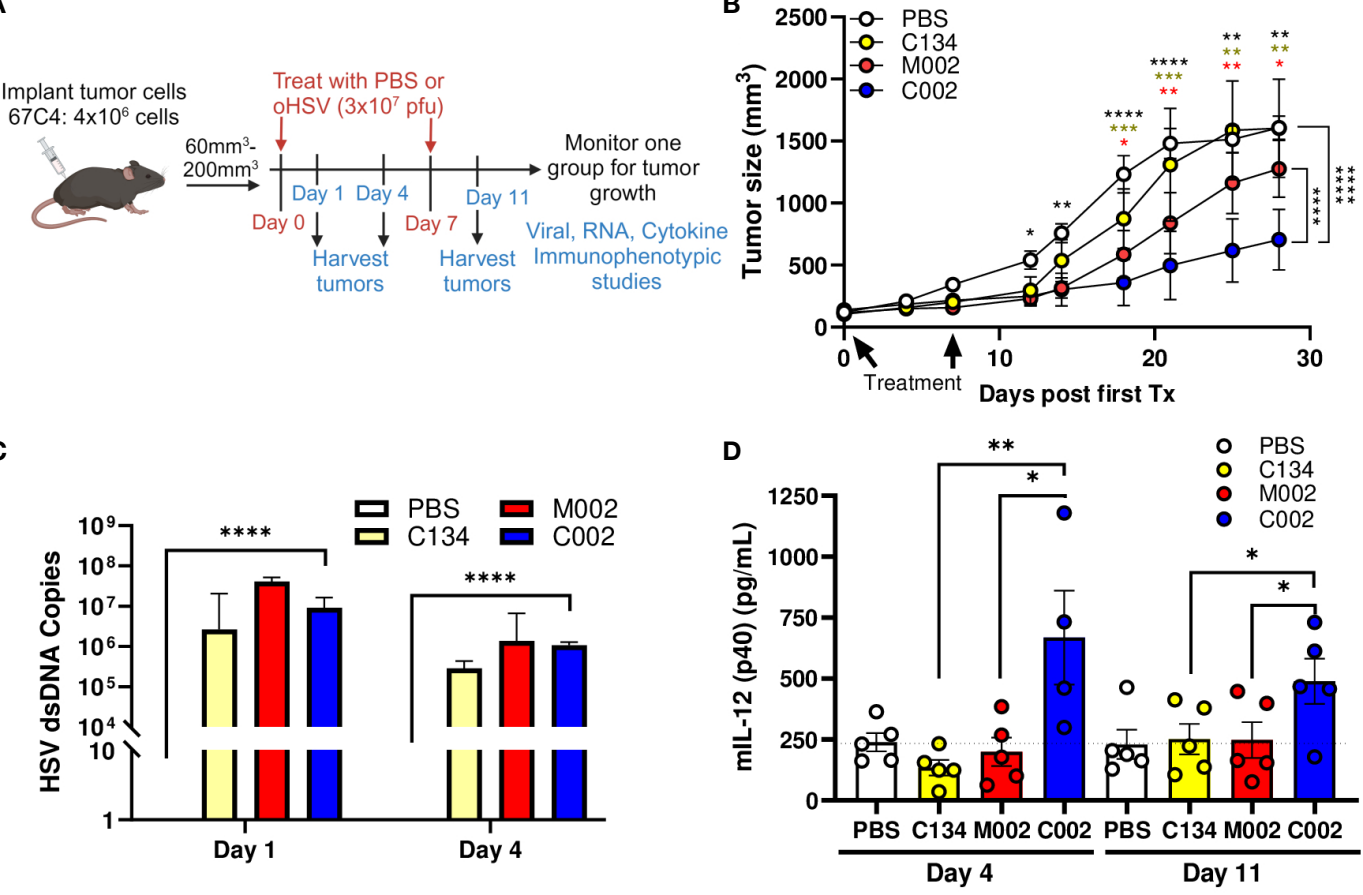
C57BL/6-based 67C-4 MPNST summary. Three- to 4-week-old C57BL/6 mice were implanted subcutaneously with 4×10^6^ cells in 50µL of phosphate buffered saline (PBS)/flank. When tumor sizes reached 60–200mm^3^, animals were randomized into treatment groups by tumor size to ensure a similar average tumor size per cohort. Tumors were then treated with saline or oHSV C134, M002, or C002 (3×10^7^ PFU in 50µL PBS)/flank intratumorally (ITu). The tumors were measured twice/week until endpoint for tumor growth studies [PBS (n=6), C134 (n=6), M002 (n=7), C002 (n=7)]. A separate cohort of mice were sacrificed at 1- and 4- days post-treatment, tumors harvested and homogenized by mechanical disruption. DNA was extracted from aliquots of tissue homogenates and qPCR-amplified for viral copies. Another aliquot of tissue homogenates were incubated with an equal volume of SDS-free RIPA lysis buffer, centrifuged, supernatants collected, and analyzed with either mouse IL-12 (p70) ELISA MAX Deluxe Set or with cytokine bead array (ProcartaPlex). **(A)** Experimental design overview schematic. **(B)** Tumor growth in saline or oHSV treated cohorts. C002 treatment significantly reduced 67C-4 tumor growth rate compared to saline (p<0.0001), C134 (p<0.0001), or M002 (p<0.0001) -treated tumors cohorts. Data were analyzed using mixed effect model. **(C)** All oHSV treatments produced similar levels of vDNA at both 1- and 4-days post treatment, and only C002 treatment produced statistically greater vDNA levels than PBS treatment (p<0.0001). **(D)** C002 produced greater IL-12 (p40) protein than the other oHSV treated cohorts when measured at Day 4 and Day 11 (D4 after second injection). Day 1: n=6/cohort, Day 4: PBS (n=5), C134 (n=5), M002 (n=5), C002 (n=4). Day 11: PBS (n=5), C134 (n=5), M002 (n=5), C002 (n=5). *p<0.05, **p<0.01, ***p<0.001, ****p<0.0001.

Next, we investigated whether C002 also maintains a therapeutic advantage over M002 in 67C-4 MPNSTs, which restrict oHSV replication in cell culture. Tumors were established in C57BL/6 mice and treated with 2 doses of PBS or oHSV (1 week apart), as performed previously (8) and summarized in the provided schematic (**Figure 3A**). Consistent with our previous results (8), the non-cytokine expressing control oHSV (C134) was ineffective in this OV-resistant sarcoma. The tumor growth results suggested that both IL-12 viruses (M002 and C002) exhibited early therapeutic activity in 67C-4 tumors, with M002 improving survival statistically compared to saline *(p=0.049)*, but the M002-treated tumors grew over time and were not significantly different from the saline-treated cohort at endpoint (*p>0.05*). In contrast, C002 reduced 67C-4 tumor growth when compared to saline (*p<0.0001*), C134 (*p<0.0001*), or M002 (*p<0.0001*) and improved survival in mice treated with saline *(p=0.0072)* or C134 *(p=0.011)* (**Figure 3B**, **Supplementary Figure 1B**).

We also measured HSV DNA levels using TaqMan qPCR assay. Similar to the B109 study, there were no significant differences in the viral DNA counts among the oHSV- treated tumor cohorts, both at day 1 and day 4 post-treatment (**Figure 3C**). These findings indicate that the observed improvement in the therapeutic efficacy of C002 cannot be attributed to enhanced viral replication.

Next, we measured IL-12 protein levels in the treated tumors to investigate if IL-12 protein production could account for the differences in anti-tumor effect between the oHSV-IL-12 viruses. IL-12 protein (p40) was measured from clarified tumor homogenates by Luminex, a cytokine bead array (**Figure 3D**). Consistent with the results from the B109 tumors, C002 significantly increased IL-12 levels than did M002 at both Day 4 (668.4pg/ml vs. 199.1pg/ml, *p=0.0227*) and Day 11 (489.4pg/ml vs. 248.2pg/ml *p=0.0443*) post-treatment in in 67C-4 tumors. IL-12 levels in C002-treated tumors were also higher than those in C134-treated tumors at both Day 4 (668.4pg/ml vs 134.5 pg/ml, *p=0.0023*) and Day 11 (489.4pg/ml vs. 251.6pg/ml, *p=0.042*).

In this study, M002 generated similar IL-12 protein amounts as the non-IL-12 oHSV- or saline-treated samples did at both time points measured. Additional assessment of IL-12 (p70) protein levels by traditional ELISA (similar to the B109 studies) confirmed that functional p70 IL-12 was consistently present in the C002 treated tumor samples (**Supplementary Figure 2A**). To evaluate if transcriptional differences between the viruses could account for these IL-12 protein changes after C002 and M002 treatment, we performed qRT-PCR, which showed abundant IL-12 transcript in the M002-treated tumors (**Supplementary Figure 2B**). In conclusion, these results demonstrate that there was reduced IL-12 cytokine produced in M002-treated 67C-4 tumors, despite increased viral recovery and mIL-12 transcription, suggesting that M002-expressed transgene was not translated into IL-12 protein.

### C002 treatment increased immune cell infiltrates within 67C-4 tumors

Next, we examined how IL-12-oHSV treatment affected tumor infiltrating immune cell populations. Mice were sacrificed at Day 11 post-initial treatment (D4 post-2^nd^ treatment), and tumor infiltrating leukocytes were analyzed by spectral flow cytometry. Phenotypic characterization was initially performed using an unsupervised approach using self-organized cluster maps (FlowSOM) from tumor infiltrating leukocyte populations isolated from the tumors using a concatenated training sample followed by individual sample analyses (Data not shown). Cluster differences were assessed and verified by traditional two-dimensional (2-D) flow cytometry analysis between the treatment cohorts to confirm these population changes. A sequential gating strategy was performed for a comprehensive phenotypic analysis of both lymphoid and myeloid subsets (**Supplementary Figure 3**).

**Figure 4:**
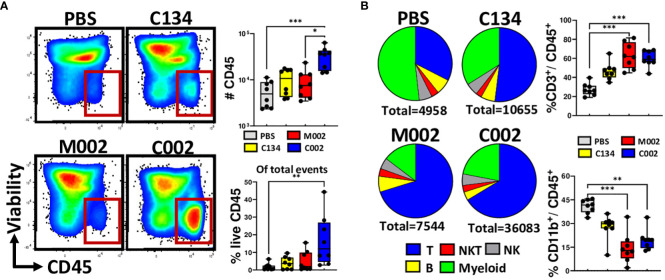
Compositions of tumor infiltrating immune cells. Tumors were treated with either PBS (n=8), C134 control (Δγ_1_34.5, IRS1; n=8), M002 (Δγ_1_34.5, mIL-12; n=8), or C002 (Δγ_1_34.5, IRS1, mIL-12; n=8). On Day 11 post-treatment (Day 4.5, post second oHSV treatment), mice were sacrificed, and tumor infiltrates isolated. Tumor infiltrating leukocytes were stained with fluorescent labelled antibodies and analyzed using spectral flow cytometry. **(A)** Representative flow plots, proportion and absolute quantity of live CD45(+) cells within the total tumor infiltrates, **(B)** Pie chart showing the percentage compositions of T-, B-, Natural killer T (NKT)-, Natural Killer (NK)- and myeloid cells within the intratumoral live CD45(+) populations. The box plots shows the differences in the frequenceis of CD3(+) T cells and CD11b(+) myeloid cells among the treatment groups. Results are presented as box and whisker plots showing the median, with 25–75 percentile range as the box and 5–95 percentiles as the whiskers. Differences in the frequencies and absolute quantities among the groups were compared using Kruskal-Wallis test with Dunn’s *post-hoc* analysis for multiple comparison. *p<0.05, **p<0.01, ***p<0.001, ****p<0.0001.

Tumors treated with C002 exhibited significantly greater CD45(+) immune infiltrates compared to those treated with PBS or M002 (**Figure 4A**). Among the treatment groups, only C002-treated tumors had increased proportions of CD45(+) cells, indicating an enhanced immune activity induced by C002-treatment. Within the CD45(+) infiltrates, both C002 and M002-treated tumors predominantly showed a CD3(+) T cell enriched microenvironment with proportional decreases in the CD11b(+) myeloid populations compared to PBS- treated tumors (**Figure 4B**). Despite their lower frequencies in the oHSV- treated tumors, we proceeded to investigate the specific myeloid cell composition within tumor infiltrates by further phenotyping CD11b(+) cells based on lineage-specific markers for monocytes, macrophages, granulocytes, and dendritic cells (**Supplementary Figures 4A-G**). While there was a significant decrease in myeloid cell frequencies in the oHSV-treated tumors, there was an increase in the absolute numbers, specifically in C002-treated tumors compared to M002 and PBS treatments (**Supplementary Figures 4A-G**). While the increase in myeloid numbers in the C002-treated group may have functional implications, it is important to note that this myeloid cell increase was linked to the greater overall CD45(+) cells abundance after C002 treatment.

### C002 treatment increased pro-inflammatory monocyte and antigen presenting cell function in 67C-4 MPNSTs

**Figure 5:**
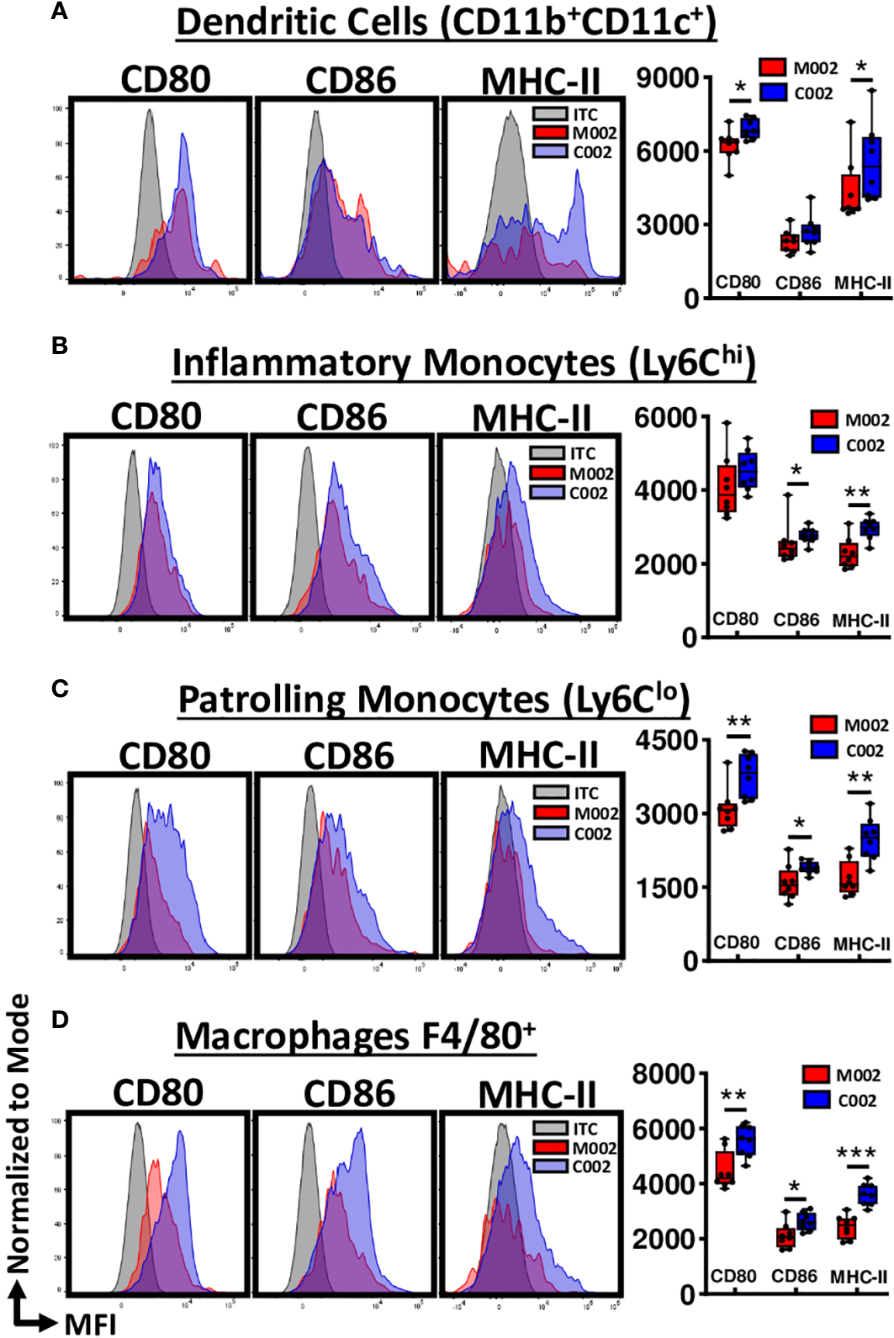
Average relative surface expression (MFI) of CD80, CD86 and MHC-II on Myeloid cells. Using flow cytometry, activation status of myeloid cells, particularly antigen-presenting cells (APCs), were assessed by quantifying the expression level of CD80, CD86 and MHC-II at the single-cell level and expressed as mean fluorescence intensity (MFI). Representative histograms and data showing the differences in MFI-CD80, MFI-CD86 and MFI-MHC-II on **(A)** CD11b(+)CD11c(+) Dendritic cells, **(B)** Ly6C^HI^ inflammatory Monocytes, **(C)** Ly6C^LO^ patrollingMonocytes, and **(D)** F4/80(+) Macrophages from M002 and C002 treated tumors. Results are presented as box and whisker plots showing the median, with 25–75 percentile range as the box and 5–95 percentiles as the whiskers. Differences in the frequencies and absolute quantities between the groups were compared using Mann-Whitney test. *p<0.05, **p<0.01, ***p<0.001, ****p<0.0001. ITC= Isotype control.

To investigate whether there were functional differences within the myeloid phenotypes associated with C002, we investigated the activation status of these myeloid cells, focusing on the upregulation of the co-stimulatory molecules, CD80 and CD86, along with MHC-II expression. These molecular signatures serve as indicators of cellular activation and effective antigen presentation. We used mean fluorescence intensity (MFI) to quantify CD80, CD86, and MHC-II expression level on a single-cell basis and found that CD11b(+) dendritic cells isolated from C002-treated tumors exhibited increased CD80 and MHC-II surface expression compared to those from M002-treated tumors (**Figure 5A**). We observed no significant changes in the expression pattern of CD11b (–) dendritic cells for MHCII or co-stimulatory markers between the two treatments (**Supplementary Figure 4H**). Next, we examined Ly6C^HI^ and Ly6C^LO^ monocytes and found CD86 and MHC-II expression was significantly upregulated in both subsets from C002-treated tumors (**Figures 5B, C**) compared to M002-treated samples. The Ly6C^LO^ patrolling monocytes also showed increased CD80 expression (**Figure 5C**) following C002 treatment. Finally, there was a significant upregulation of all three molecules (MHCII, CD80, &CD86) in F4/80(+) macrophages from C002-treated tumors compared to M002-treated tumors (**Figure 5D**). These macrophages also produced greater TNF-α after C002-treatment compared to M002-treated tumors (**Supplementary Figure 4I**). These findings show that there was not only an increase in the number of monocytes and antigen presenting cells (APCs) but that there were some functional differences with increased costimulatory (CD80, CD86) and MHC-II expression following C002 treatment.

### C002-treatment increased polyfunctional CD4(+) T cell effectors in 67C-4 tumors

Based upon the increased expression of co-stimulatory molecules (CD80 and CD86) and MHC-II upregulation differences in APCs isolated from C002-treated tumors, we hypothesized that the upregulation of these surface repertoires in APCs lead to an amplified T cell, particularly CD4(+) cell response. To test this, we examined lymphoid cell dynamics (numbers, activation status, and memory phenotypes), from both the PBS- and oHSV-treated tumors.

**Figure 6:**
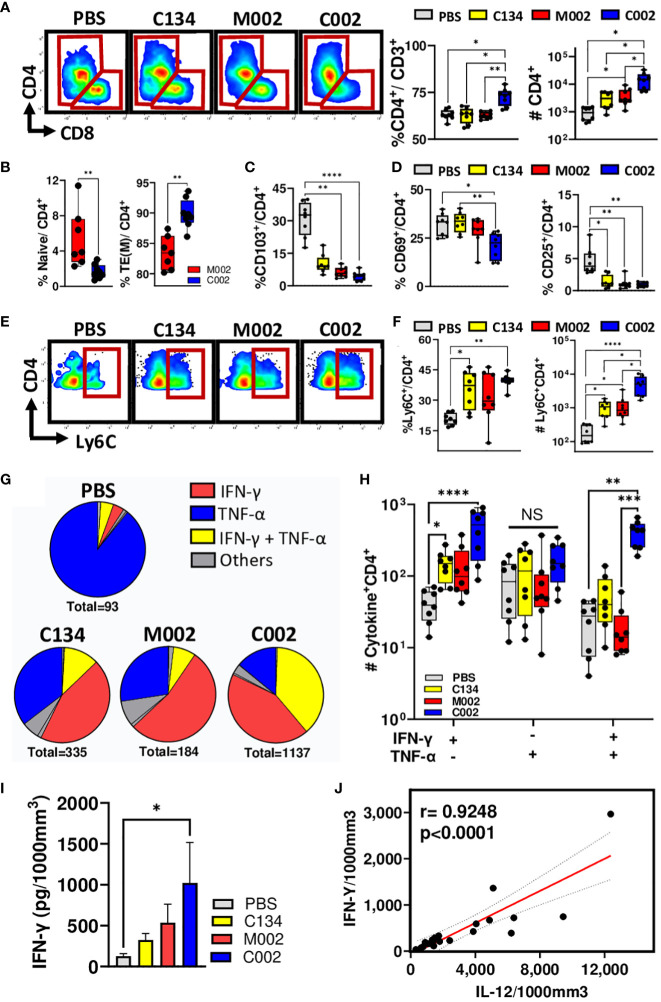
Tumor-infiltrating lymphocytes (TIL) frequency, memory subsets, activation status, and functions. PBS (n=8) or oHSVs treated tumors (C134, n=8; M002, n=8 and C002, n=8) were harvested on Day 11 post-treatment. Tumor infiltrating leukocytes were stained with fluorescent labelled antibodies for surface markers, intracellular cytokines (TNF-α, IFN-γ, IL-4, IL-17, IL-21 and Granzyme B) and transcription factor (FoxP3), and analyzed using spectral flow cytometry. **(A)** Representative flow plots, proportion [within the CD3(+) population] and absolute quantity of CD4(+) T-helper (Th) cells among the groups. **(B)** Proportions of CD44(-)CD62L(+) Naïve and CD44(+)CD62L(-) Effector/Effector memory phenotypes within the CD4(+) populations. **(C)** Proportions of CD103(+) tissue resident memory (TRM) phenotypes among CD4(+) T cells. **(D)** Activation status of CD4(+) T cells were assessed by quantifying CD69 and/or CD25 surface expression. The graph shows the proportions of CD69(-)CD25(+)and CD69(-)CD25(+)cells among total CD4(+) T cells. **(E)** Representative flow plots, **(F)** proportions (within the CD4+ population) and absolute quantity of Ly6C expressing CD4+ T cells among the PBS and oHSV treated cohorts. **(G)** Pie chart showing the proportions of CD4(+) T cells expressing either cytokines, TNF-α or IFN-γ alone or in combinations. **(H)** Differences in the frequencies of single (TNF-α or IFN-γ) or multiple (TNF-α and IFN-γ) expressing CD4(+) T cells among the treated cohorts. **(I, J)** Intratumoral level of IFN-γ **(I)** and IL-12 was measured using cytokine bead array and depicted as picograms (pg) of cytokines present in 1000mm^3^ of the tumor volume. **(J)** Intratumoral IL-12 levels correlated with IFN-γ levels using non-parametric Spearman correlation. Results are presented as box and whisker plots showing the median, with 25–75 percentile range as the box and 5–95 percentiles as the whiskers. Figure I is represented as bar diagram for mean with standard error of mean (SEM). Differences in the frequencies and absolute quantities among the groups were compared using Kruskal-Wallis test with Dunn’s *post-hoc* analysis for multiple comparison. Mann-Whitney test was performed to assess the differences in the frequencies between two groups **(B)**. *p<0.05, **p<0.01, ***p<0.001, ****p<0.0001.

Consistent with our and others’ past studies (8, 22–24), virotherapy had its greatest impact on the T cell population and produced no significant change in CD19(+) B cell frequencies when compared to mock-treated cohorts (**Supplementary Figure 5A**). Both M002 and C002 treatments significantly increased CD3(+) T cell frequencies and numbers when compared to PBS-treated tumors (**Figure 4B**). Notably, there were increases in the αβ-T phenotype population, as evidenced by the observed decrease in γδ-T cell proportions (**Supplementary Figure 5B**). In addition, CD3(+)NK1.1(+) Natural Killer T (NKT) cells and CD3(-) NK1.1(+) NK cell frequencies remained unchanged (**Supplementary Figures 5C, D**), highlighting the stability of this distinct T cell subset within the total T cell population. Further phenotyping of T cells revealed that C002 tumor treatment increased both CD4(+) T cell frequency and absolute number (**Figure 6A**), with a concomitant decrease in the CD8(+) frequency (**Supplementary Figure 5E**), resulting in an increased CD4: CD8 ratio (**Supplementary Figure 5F**).

Next, to characterize the CD4(+) population, we examined CD62L/CD44 surface expression and phenotyped their naïve, memory or effector status. We characterized these cells as CD44(-)CD62L(+) naïve, CD44(+)CD62L(+) central memory (TCM), or CD44(+)CD62L(-) effector/effector memory (TEM) and expressed them as a percentage of total CD4(+) T cells. Tumors treated with C002 had a decrease in naïve populations (**Figure 6B**) with a simultaneous increase in effector/effector memory populations (**Figure 6B**). There was no significant difference in the frequency of central memory populations between C002- and M002-treated cohorts (**Supplementary Figure 5G**). Further investigation into the tissue residency of memory CD4(+) T cells, using integrin αE (CD103) as markers of tissue-resident memory (TRM) cells, revealed significant reduction of TRM cells in both C002- and M002-treated tumors (**Figure 6C**). Surprisingly, using the canonical CD69 and CD25 as markers of T-cell activation, we detected a reduction in the composition of CD69(+)CD25(-), CD69(-)CD25(+), and CD69(+)CD25(+) activated CD4 cell phenotypes (**Figure 6D**, **Supplementary Figure 5H**). In parallel, we also characterized the CD8(+) T cytotoxic populations. No significant changes were noted for naïve and memory populations between C002- and M002-treated tumors (**Supplementary Figures 5I, J**), suggesting that CD8(+) T cells in C002-treated tumors may have a less active role in maintaining the anti-tumor response at the time point sampled in this tumor model.

Because we detected an increase in effector/effector memory CD4(+) T cell frequency within C002-treated tumors, we next examined the functional potential of these intratumoral CD4 T cells. Consistent with recent studies that have highlighted the pro-inflammatory nature of Ly6C(+)CD8(+) T cells (25, 26), we identified a significant increase of Ly6C(+), CD8(+) T cells in C002-treated tumors relative to PBS-treated tumors (**Supplementary Figures 5K-M**). This finding led us to investigate Ly6C expression on the CD4(+) T cells in the tumors where we anticipated a similar pro-inflammatory phenotype change akin to the Ly6C(+)CD8(+) T cells. The results showed both increased frequency and absolute counts of Ly6C(+)CD4(+) T cells in C002-treated tumors (**Figures 6E, F**). The role of these cells in tumor immunity and if they are unique to virotherapy treatment will require further investigation. Next, we assessed the anti-inflammatory T cells in the tumor microenvironment by quantifying FoxP3(+) regulatory T cells (Tregs). As anticipated, oHSV treatment decreased Treg infiltrates in the tumor (**Supplementary Figure 5N**). Collectively, these results indicate a shift towards a more pro-inflammatory with reduced immunosuppressive microenvironment in C002-treated tumors.

Given the increase in pro-inflammatory CD4(+) T cells after oHSV treatment, we next analyzed IFN-γ, TNF-α, IL-4, IL-17A, and IL-21 intracellular expression within the CD4(+) tumor infiltrates. CD4(+) T cells expressing IL-17 and IL-21 constituted less than 0.89 ± 0.11% of all cytokine-producing CD4(+) T cells, indicating minimal involvement of Th17 and T- follicular helper (Tfh) in the tumor microenvironment (**Supplementary Figures 5O, P**). Additionally, we detected a significant decrease in IL-4(+) Th2 cells in M002-treated tumors compared to PBS-treated tumors (**Supplementary Figure 5Q**). The majority of CD4(+) T cells expressed either TNF-α and/or IFN-γ, indicating a T-helper 1 (Th1) subset predominance in the tumor infiltrates. Notably, oHSV treatment shifted the cytokine dynamics from TNF-α to IFN-γ, resulting in increased frequencies of single (IFN-γ) or double (IFN-γ and TNF-α) cytokine-producing cells (**Figure 6G**). Among the oHSV-treated cohorts, C002-treated tumors exhibited the highest number of IFN-γ and/or TNF-α producing CD4(+) T cells (**Figure 6H**), suggesting a skew in cellular response towards an IFN-γ expressing polyfunctional Th1 phenotype. Consistent with the flow data, C002-treated tumors also exhibited higher IFN-γ protein levels than did PBS-treated tumors (**Figure 6I**) that correlated with the level of IL-12 protein in the tumors (**Figure 6J**).

We detected comparable CD45+ tumor infiltrating lymphocyte populations between the two IL-12-oHSV-treated cohorts in B109 tumors (**Supplementary Figure 6A**). C002-treated tumors showed fewer B cells in frequency and a smaller CD11b+ myeloid cell population than did M002-treated tumors (**Supplementary Figures 6B, C**). Notably, this reduction in the myeloid and B-cell populations was accompanied by a compensatory increase in T- cell populations (**Supplementary Figure 6D**). Similar proportions of CD4(+) and CD8(+) T cells were present in M002 and C002 (oHSV-IL-12)-treated B109 tumors (**Supplementary Figure 6E**). However, T cells differed in memory and functional aspects between tumors treated by each IL-12 oHSV. C002-treated tumors contained a greater proportion of CD8(+) T cells that were effector memory cells (**Supplementary Figure 6F**) and expressed more granzyme-B (**Supplementary Figure 6G**) than M002-treated samples did, similar to that observed in the 67C-4 tumors (**Supplementary Figure 4I**). Furthermore, the majority of CD4(+) T cells in C002-treated tumors were effector memory cells (**Supplementary Figure 6H**) and showed increased IFN-γ expression compared to M002-treated tumors (**Supplementary Figure 6I**).

In summary, our studies show that despite similar transcriptional activity, a translationally optimized IL-12 expressing oHSV (C002) produced greater IL-12 cytokines, demonstrated greater IL-12 related immune functional activity, and improved the MPNST therapeutic response compared to another IL-12 expressing oHSV (M002).

## Discussion

MPNSTs are challenging tumors for oncolytic herpes simplex virus (oHSV) treatment (7, 8). The tumors restrict viral replication and oHSV-associated immune therapeutic activity (7, 8). To address these issues, we sought ways to improve immune mediated activity using virus-based cytokine expression and examined two immune competent murine MPNST models (B109 and 67C-4) that differ in their oHSV susceptibility (7). Our prior studies (6, 7) highlighted differences in MPNST susceptibility to infection and gene expression *in vitro* and focused on human MPNSTs. However, a limitation of human tumor studies lies in its dependence on using immunocompromised animal models for evaluating virotherapy efficacy. This approach eliminates an important component: the immune- mediated therapeutic effects. We therefore used immune syngeneic immune-competent syngeneic murine models to examine the oHSV mediated immune landscaping within the tumor microenvironments.

This study highlights differences between oncolytic HSVs, including those expressing the same immune active murine Interleukin-12 (mIL-12) cytokine, in these murine sarcomas. Both M002 (Δγ_1_34.5, mIL-12) and C002 (Δγ_1_34.5, IRS1, mIL-12) were constructed using the same targeting plasmid and therefore share the same promoter and IL-12 bicistronic cassette. Our results confirmed that both C002 and M002 produced similar IL-12 transcript levels in the treated tumors but only C002 increased IL-12 protein levels. We previously showed that C134 evades dsRNA-activated translational arrest, improving late viral gene expression, in contrast to early generation (Δγ_1_34.5) oHSVs (13, 14). To our knowledge, this is the first time that a virus with improved late gene expression has been shown to improve virus encoded protein production *in vivo*. These IL-12 results suggest that C134-based oHSVs improve therapeutic transgene expression in the *in vivo* environment when compared to an early generation IL-12 oHSV (M002).

C002 (Δγ_1_34.5, IRS1, mIL-12) and M002 (Δγ_1_34.5, mIL-12) also induced unique immune effector changes in treated tumors, reflective of the dissimilar IL-12 protein expression. C002-treated tumors exhibited increased absolute myeloid (CD11b+) populations, primarily driven by infiltrating monocytes (Ly6C+), macrophages (F4/80+), and dendritic cells (CD11c+). We observed pro-inflammatory changes (Ly6C^HI^) and, possibly, improved antigen presentation (MHC-II, CD80 and CD86) within these populations, particularly in the CD11b(+)CD11c(+) myeloid-derived conventional dendritic cells (cDCs). The macrophages adopted an M1-like phenotype, contributing to an overall pro-inflammatory milieu induced by C002.

C002 treatment also increased the tumor infiltration of CD44(+)CD4(+) T cells, suggesting a shift from naïve to effector/effector memory T cell populations. Further, the absence of CD103 expression as tissue resident marker in these CD4(+) T cells implies they may have migrated from nearby draining lymph nodes after being induced by viral presence in the tumors. At this stage of the study, it is still unclear whether these cells, potentially antigen-specific and influenced by IL-12-related MHC-II presentation changes, are licensed at the lymph node or elsewhere outside of the tumor and whether these cells have other anti-tumor functional activity.

Characterization of CD4(+) T cells demonstrated a predominant Th1 phenotype within C002-treated tumors, evidenced by increased IFN-γ expression and a proportional decrease in TNF-α production. Intriguingly, our analysis did not detect Th2, Th17, Tfh or Tregs, indicating a skewed differentiation favoring pro-inflammatory Th1 responses within the tumor microenvironment. Of particular interest was the induction of Ly6C expression on CD4(+) T cells by C002. Ly6C, a protein belonging to the Ly-6 superfamily and often used as a monocyte marker, has traditionally been linked to IFN-γ expressing pro-inflammatory characteristics, especially in CD8(+) cells during bacterial lung infection and acute lung injury in mice (25, 26). Additionally, Ly6C is recognized for homing CD8(+) T cells to secondary lymphoid tissues (27). Similarly, previous studies in LCMV infection have shown that Ly6C^HI^ CD4(+) T cells produced higher levels of IFN-γ and granzyme B compared to Ly6C^LO^ cells (28). In our investigation, we identified that C002 induced the expression of Ly6C on the surface of CD4(+) T cells, signifying a pro-inflammatory state. The functional relevance of Ly6C(+)CD4(+) T cells in the context of anti-tumor activity in our study remains unknown at this stage. However, this finding prompts further exploration in future studies to decipher the specific role of this population and its potential implications for the overall anti-tumor immune response induced by C002 treatment.

With the increased population of effector CD4(+) T cells in C002-treated tumors, we anticipated an increased activation state within these cells. While our analysis showed heightened expression of the early activation marker CD44, we did not observe significant changes in other activation markers such as CD69 and CD25. The unexpected absence of CD69 and CD25 in C002-induced CD4(+) T cells raises questions about the optimal time for conducting immunophenotyping of tumor infiltrates to capture the nuanced changes in T cell activation. Performing immunophenotyping at multiple timepoints post-treatment in future studies could provide valuable insights into the evolving activation stages induced by these oHSVs.

These studies also highlight the importance of *in vivo* oHSV activity assessments. Virotherapy involves a complex anti-tumor response encompassing both direct viral activity within the infected cancer cell and a complex immune-mediated anti-tumor bystander effect. It is not surprising that *in vivo* studies are necessary to assess this complex tumor-associated immune therapeutic activity. These studies, however, also highlight how direct viral functions (replication and gene expression) can also differ between *in vitro* and *in vivo* studies. *In-vitro*, B109 cells supported both M002 and C002 gene expression, IL-12 cytokine protein production, and viral replication (10^6^–10^7^ PFU). However, the B109 MPNST tumors behaves similarly to the more resistant 67C4 tumors. Viral recovery was similar across both tumor models for the oHSV tested *in vivo*. Furthermore, the B109 tumors were more restrictive to the early generation IL-12 oHSV (M002) in terms of IL-12 production. Although B109 tumors were less aggressive than 67C-4 tumors, they were resistant to C134 or M002 therapy. Neither of the murine sarcomas (B109 nor 67C-4) supported robust oHSV replication as occurs in human tumor models (14). The early generation and next generation oHSVs replicated similarly, producing equivalent IL-12 transcript, but only the C134-based virus (C002) increased IL-12 cytokine protein levels *in vivo.* The enhanced IL-12 production in C002-treated tumors, however, did not suppress viral replication.

Interestingly, differences in IL-12 protein production, absolute increases in immune infiltrates, and functional changes related to IL-12 production (T cell and APC activity), did not associate with viral recovery differences between the two IL-12 viruses. While speculative, it is possible that the early IFN response and the restrictive murine tumor cell environment play greater roles in restricting viral replication than does the later adaptive immune-mediated antiviral response in these immune-competent murine models. This highlights a limitation to this study that HSV is a human virus, and viral gene expression and replication is less efficient in murine cells. This virus vs host difference may have exaggerated the observed decrease in cytokine production in the early generation oHSV-treated tumors in this study.

Both cytokine viruses require further *in vivo* evaluation for cytokine expression levels and replication in human tumors. Human-based cell studies and *in vivo* xenograft studies may reveal improved cytokine expression, replication, and spread from early generation oHSVs that are not evident from these syngeneic studies. Unfortunately, xenograft studies are imperfect and do not permit a fully integrated immune competent model system with the necessary type I IFN response to fully assess oHSV-related IL-12 activity.

It is also pertinent to address potential concerns regarding the use of IL-12 as an immunotherapy considering its severe adverse effects when administered systemically in early clinical trials in the 1990s (29, 30). In the decades since, however, IL-12 has remained an enticing target to scientists and subsequently been demonstrated to be safe to use and tolerable when administered locally rather than systemically, with some preclinical results advancing to phase I clinical trials (19, 31). One of these therapies includes the aforementioned M032, an (h)IL-12-expressing HSV-1 that is the human equivalent of M002, which has concluded a dose-escalating phase I clinical trial (NCT02062827) designed to test the safety and tolerability of intratumoral administration in patients with recurrent or progressive malignant glioma. Intratumoral M032 had an acceptable adverse-event profile, and no dose-limiting toxicity occurred at the greatest dose (32).

It is possible that cytokine production of the early generation oHSV may be sufficient in human tumors, as preliminary results from the M032 phase I clinical trial reveal positive responses in some patients (32). Alternatively, it is also possible that the improved cytokine production of the next generation IL-12 oHSV (C002) may be too robust in human tumors, leading to cytokine release syndrome-related morbidity that is poorly predicted by the murine models. We are investigating several approaches in ongoing studies to advance these cytokine viruses using FDA approved medications that we anticipate could be readily incorporated into future oHSV-IL-12 clinical trials and may allow more refined control of the cytokine production *in vivo*.

One advantage of these studies was the extensive TME cytokine and immune cell phenotypic characterization. By using a full spectrum flow cytometry technique and quantifying tumor-associated cytokine production, we were able to analyze both adaptive and innate TIL changes from the limited numbers of tumor infiltrating leukocytes and identify microenvironment changes post-treatment in the MPNSTs. By incorporating intracellular cytokine staining, we were also able to further characterize T cell functional response changes in the oHSV-treated tumors. We focused on timepoints from past studies where we observed adaptive immune cell changes (4–7d post treatment), but this may have biased the results toward adaptive immune changes. While these studies also detected myeloid cell response changes between oHSV-treated samples like MHCII CD80/86 upregulation in the monocyte, macrophage, and myeloid DC populations, it is possible that other myeloid- and innate cell-associated changes are present at earlier timepoints post-oHSV treatment that these studies did not detect. Additionally, functional studies will be necessary in the future, despite our current findings, which are indicative of APC antigen presentation, increases in pro-inflammatory and patrolling monocytes, and increases in polyfunctional cytokine producing CD4 infiltrates. Functional studies will also allow us to assess whether C002 improves the response of tumor antigen-specific CD4(+) T cells. Lastly, a limitation with oHSV treatment is that anti-tumor responses to treatment may not be uniform. In the B109 tumor model, some tumors were cleared while others grew irrespective of treatment. In the aggressive 67C-4 MPNST model, oHSVs did not produce any cures and only had transient tumor suppressive activity but required repeated dosing. In human studies we anticipate that repeated dosing may be required.

## Data availability statement

The original contributions presented in the study are included in the article/**Supplementary Material**. Further inquiries can be directed to the corresponding author.

## Ethics statement

The animal study was approved by Institutional Animal Care and Use Committee (IACUC) at Nationwide Children’s Hospital. The study was conducted in accordance with the local legislation and institutional requirements.

## Author contributions

YK: Data curation, Formal analysis, Investigation, Methodology, Project administration, Validation, Writing – original draft, Writing – review & editing. US: Conceptualization, Investigation, Methodology, Supervision, Writing – original draft, Writing – review & editing. DK: Writing – original draft, Writing – review & editing. IH-A: Writing – original draft, Writing – review & editing. JH: Writing – original draft, Writing – review & editing. AM: Writing – original draft, Writing – review & editing. XM: Formal analysis, Software, Validation, Writing – original draft, Writing – review & editing. TC: Writing – original draft, Writing – review & editing. JM: Writing – original draft, Writing – review & editing. KC: Conceptualization, Formal analysis, Funding acquisition, Investigation, Resources, Supervision, Writing – original draft, Writing – review & editing. RD: Formal analysis, Investigation, Supervision, Writing – original draft, Writing – review & editing.

## Supplementary material

SUPPLEMENTARY Figure 1 Kaplan-Meier survival summary of C57BL/6-based MPNSTs. Three- to four-week-old C57BL/6 mice were subcutaneously implanted with tumor cells in 50 µL of PBS per flank. Upon reaching tumor sizes of 64-600 mm^3^ (B109) or 60-200mm^3^ (67C-4), animals were randomized. Tumors were treated intratumorally (ITu) with saline or oHSV (C134, M002, or C002) at a dosage of 3×10^7^ PFU in 50 µL PBS per flank. Tumor measurements were taken twice per week until the study's endpoint for tumor growth assessment. Death events were recorded. Tumors exceeding 1250 mm^3^ were also considered indicative of animal death to adhere to our IACUC euthanasia guidelines regarding tumor growth. Kaplan-Meier survival analysis was conducted using GraphPad Prism v10.1.0 **(A)** C002 provided the highest survival benefit compared to all other treatments in B109 tumor-bearing mice [Log Rank (Mantel-Cox) test: C002 vs Saline, *p=0.0037*; C002 vs C134, *p=0.0011*; and C002 vs M002, *p=0.0126*]. **(B)** Mice bearing 67C-4 tumors treated with C002 survived longer than did mice treated with Saline or C134, while mice treated with M002 differed in survival statistically from those treated with Saline [Log Rank (Mantel-Cox) test: C002 vs Saline, *p=0.0072*; C002 vs C134, *p=0.0108*; C002 vs M002, *p=0.0898*; M002 vs Saline, p=0.0498]. *p<0.05, **p<0.01, ***p<0.001, ****p<0.0001.

SUPPLEMENTARY Figure 2 IL-12 (p70) protein and transcript levels in C57BL/6-based 67C-4 MPNSTs. Three- to 4-week-old C57BL/6 mice were implanted subcutaneously with 4×10^6^ cells in 50µL of phosphate buffered saline (PBS)/flank. When tumor sizes reached 60-200mm^3^, animals were randomized into treatment groups by tumor size to ensure a similar average tumor size per cohort. Tumors were then treated with saline or oHSV C134, M002, or C002 (3×10^7^ PFU in 50µL PBS)/flank intratumorally (ITu). Mice were sacrificed at 1- and 4- days post-treatment, tumors harvested and homogenized by mechanical disruption. RNA was extracted from aliquots of tissue homogenates, converted to cDNA and qPCR performed to quantify IL-12 RNA levels. Another aliquot of tissue homogenates were incubated with an equal volume of SDS-free RIPA lysis buffer, centrifuged, supernatants collected, and analyzed with either mouse IL-12 (p70) ELISA MAX Deluxe Set. **(A)** C002 produced greater levels of IL-12 (p70) protein than all other virus cohorts produced at both 1- and 4-days following first virus treatment in 67C-4 flank tumors detected by ELISA. **(B)** M002-treated tumors contain abundant mIL-12 transcript levels, which are similar to or greater than C002-treated tumors at both 1- and 4-days post first virus treatment.

SUPPLEMENTARY Figure 3 Gating strategies for flow cytometric analysis of myeloid and lymphoid compositions in tumors. Tumors were treated with either PBS (n=8), C134 control (Δγ_1_34.5, IRS1; n=8), M002 (Δγ_1_34.5, mIL-12; n=8), or C002 (Δγ_1_34.5, IRS1, mIL-12; n=8). On Day 11 post-treatment (Day 4, post second oHSV treatment), mice were sacrificed, and tumor infiltrates isolated. Tumor infiltrating leukocytes were stained with fluorescent labelled antibodies and analyzed using spectral flow cytometry. In the gating strategy, debris exclusion and leucocyte selection were initiated using forward scatter and side scatter area. Viable CD45(+) cells were identified through gating based on their low expression of Zombie NIR viability stain and high expression of CD45. Within the live CD45(+) gate, B cells (CD19+) were gated. Among CD19(-) population, CD3(+) cells were differentiated from CD49b(+) cells. NK cells were gated among CD3(-)CD49b(+) cells and recognized as NK1.1(+) expressing cells. Among CD3(+) cells, Natural Killer T (NKT) and T cells were differentiated based on their expression of NK1.1. T cells with CD3(+)NK1.1(-) expression were further gated for CD4(+) T helper (Th) or CD8(+) T cytotoxic (Tc) cells. CD4(+) T cells were differentiated as regulatory T cells (Tregs) based on their expression of FoxP3(±)IL-10 expression. CD4(+) or CD8(+) T cells were individually gated for their surface expression of Ly6C, CD44, CD62L, CD49b, CD103, CD25 and CD69 as well as for their intracellular cytokines (TNF-α, IFN-γ, IL-4, IL-17A, IL-21 and Granzyme B for CD4; IFN-γ and Granzyme B for CD8) expression. Combination of CD44 and CD62L expressions were used to identify naïve (CD44-CD62L+), central memory (TCM; CD44+CD62L+) and effector/ effector memory (TE/TEM; CD44+CD62L-) populations. Likewise, CD49b(±)CD103 expression was used to characterize tissue resident memory T (TRM) cells. Activation of T cells were assessed through their expression of CD25(±)CD69. Sequential gating was performed among CD19(-)CD3(-)CD49b(-) populations to identify myeloid cell populations. Neutrophils or granulocytic myeloid derived suppressor cells (gMDSC) were defined based on their expression of CD11b(+)Ly6G(+) cells. Of the remaining populations, CD11b(+)Siglec-5(+) populations were identified as eosinophils. Within the Siglec5 negative cells, CD11c(+) dendritic cells were quantified. Macrophages were characterized as F4/80(+) cells among CD11b(+)Ly6G(-)Siglec-5(-)CD11c(-) populations. Lastly, Ly6C expression within the CD11b(+)Ly6G(-)Siglec-5(-)CD11c(-)F4/80(-) populations was calculated to define monocytes or monocytic MDSC (mMDSC). We defined monocytes as “inflammatory” with their high expression of Ly6C (Ly6C^HI^) or nonclassical “Patrolling” with low expression of Ly6C (Ly6C^LO^). The frequencies of specific marker-expressing cell populations were expressed as percentages of their parent population or of total CD45(+) cells.

SUPPLEMENTARY Figure 4 Myeloid compositions of 67C-4 tumor infiltrates. Tumors were treated with either PBS (n=8), C134 control (Δγ_1_34.5, IRS1; n=8), M002 (Δγ_1_34.5, mIL-12; n=8), or C002 (Δγ_1_34.5, IRS1, mIL-12; n=8). On Day 11 post-treatment (Day 4, post second oHSV treatment), mice were sacrificed, and tumor infiltrates isolated. Tumor infiltrating leukocytes were stained with fluorescent labelled antibodies and analyzed using spectral flow cytometry. Sequential (sub-) gating was performed to identify granulocytes, eosinophils, dendritic cells, macrophages and monocytes within the tumor infiltrates and the frequenceis were expressed as percentage composition of CD45(+) cells. Representative flow plots, frequenceis and absolute quantities of **(A)** Ly6G(+) granulocytes/granulocytic myeloid derived suppressor cells (gMDSCs), **(B)** Ly6G(-)Siglec-F(+) eosinophils, **(C)** Ly6G(-)Siglec-F(-)CD11c(+) dendritic cells, **(D)** Ly6G(-)Siglec-F(-)CD11b(+)CD11c(-)F4/80(+) macrophages, and **(E)** Ly6G(-)SiglecF(-)CD11b(+)CD11c(-)F4/80(-)Ly6CHI inflammatory monocytes among the groups. **(F)** Comparaision of frequency and absolute quantity of Ly6G(-)SiglecF(-)CD11b(+)CD11c(-)F4/80(-)Ly6CLO patrolling monocytes among the treatment groups. **(G)** Frequencies and absolute quantities of CD11b(+)CD11c(+) and CD11b(-)CD11c(+) dendritic cells. **(H)** Mean fluorescence intensity (MFI) of CD80, CD86 and MHC-II expression on CD11b(-)CD11c(+) dendritic cells between M002 and C002 treated tumors. **(I)** Differences in the TNF-α expressing CD4+ T cells between M002 and C002 treated tumors.

SUPPLEMENTARY Figure 5 Characterization of Immune infiltrates among saline and oHSV treated 67C-4 tumors. PBS (n=8) or oHSVs (C134, n=8; M002, n=8 and C002, n=8) treated tumors were harvested on Day 11 post-treatment. Tumor infiltrating leukocytes were stained with fluorescent labelled antibodies and analyzed using spectral flow cytometry. **(A)** Proportions of CD19(+) B cells among CD45(+) infiltrates. **(B)** Proportions of γδ T cells within total T cells. **(C)** Proportions of natural killer T (NKT) cells and **(D)** NK cells within CD45(+) infiltrates. **(E)** Proportions of CD8(+) cytotoxic T (Tc) cells among total T cells. **(F)** CD4:CD8 ratio within intratumoral T cells. **(G)** Proportions of CD44(-)CD62L(+) naïve, CD44(+)CD62L(+) central memory (TCM) and CD44(+)CD62L(-) effector/ effector memory (TE/TEM) CD4(+) T cells. **(H)** Frequencies of CD69(+)CD25(+) activated CD4(+) T cells. **(I)** Proportions of naïve, central memory (TCM) and effector/ effector memory (TE/TEM) CD8(+) T cells. **(J)** Frequencies of naïve and TE/TEM CD8(+) T cells between C002 and M002 treated tumors. **(K)** Representative flow plot showing differences in the Ly6C expression on CD8(+) T cells among the treatement groups. **(L)** Frequencies and absolute quantities **(M)** of Ly6C(+)CD8(+) T cells. **(N)** Frequenceis of CD4+FoxP3+ regulatory T cells (Tregs). **(O)** Frequenceis of IL-17A expressing T helper 17 (Th17) cells and **(P)** IL-21 expressing T- follicular helper (Tfh), and **(Q)** IL-4 expressing Th2 cells within CD4(+) cells. Results are presented as box and whisker plots showing the median, with 25–75 percentile range as the box and 5–95 percentiles as the whiskers. Differences in the frequencies and absolute quantities among the groups were compared using Kruskal-Wallis test with Dunn’s post-hoc analysis for multiple comparison. Mann-Whitney test was performed to assess the differences in the frequencies between two groups **(J)**. *p<0.05, **p<0.01, ***p<0.001, ****p<0.0001.

SUPPLEMENTARY Figure 6 Characterization of Immune infiltrates among saline and oHSV treated B109 tumors. B109 tumors were treated with either M002 (n=4), or C002 (n=4). On Day 5 post-treatment, mice were sacrificed, and tumor infiltrates isolated. Tumor infiltrating leukocytes were stained with fluorescent labelled antibodies and analyzed using spectral flow cytometry. **(A)** Absolute numbers of tumor-infiltrating CD45(+) immune cells between the treatment groups. **(B)** Pie-chart showing the percentage composition of immune cells types within the treated tumors. **(C)** Frequenceis of intratumoral CD3(-)CD19(+) B cells and **(D)** CD3(+)CD19(-) T cells between the groups. **(E)** Ratio of T-helper and T-cytotoxic (CD4:CD8). **(F)** Proportion of CD44(+)CD62L(-) effector/effector memory CD8(+) T cells. **(G)** Frequencies of Granzyme-B (GzB) expressing CD8(+) T cells between the treatment groups. **(H)** Proportion of CD44(+)CD62L(-) effector/effector memory CD4(+) T cells. **(I)** Frequencies of Interferon-γ (IFN-γ) expressing CD4(+) T cells between the treatment groups. Differences in the frequencies and absolute quantities between the M002- and C002- treated tumors were compared using Mann-Whitney upaired t-test. *p<0.05, **p<0.01, ***p<0.001, ****p<0.0001.
