# Supplementary figures and images for: Enhanced IL-12 transgene expression improves oncolytic viroimmunotherapy
Source: Front Immunol. 2024 Jun 4;15:1375413. doi: 10.3389/fimmu.2024.1375413 (PMC11184146; doi:10.3389/fimmu.2024.1375413)

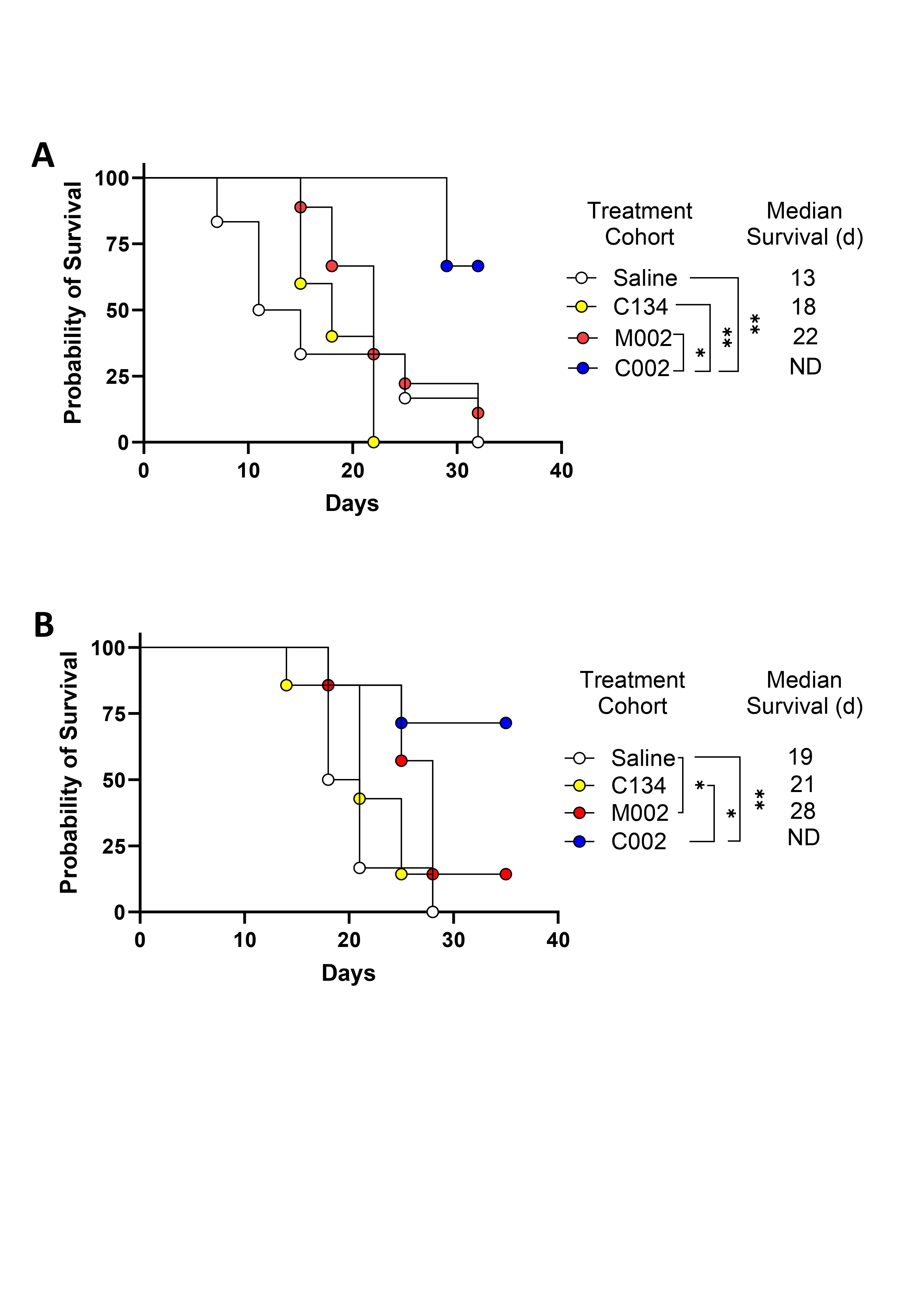

Supplement: SUPPLEMENTARY Figure 1 — Kaplan-Meier survival summary of C57BL/6-based MPNSTs. Three- to four-week-old C57BL/6 mice were subcutaneously implanted with tumor cells in 50 µL of PBS per flank. Upon reaching tumor sizes of 64-600 mm3 (B109) or 60-200mm3 (67C-4), animals were randomized. Tumors were treated intratumorally (ITu) with saline or oHSV (C134, M002, or C002) at a dosage of 3×10^7^ PFU in 50 µL PBS per flank. Tumor measurements were taken twice per week until the study's endpoint for tumor growth assessment. Death events were recorded. Tumors exceeding 1250 mm3 were also considered indicative of animal death to adhere to our IACUC euthanasia guidelines regarding tumor growth. Kaplan-Meier survival analysis was conducted using GraphPad Prism v10.1.0 (A) C002 provided the highest survival benefit compared to all other treatments in B109 tumor-bearing mice [Log Rank (Mantel-Cox) test: C002 vs Saline, p=0.0037; C002 vs C134, p=0.0011; and C002 vs M002, p=0.0126]. (B) Mice bearing 67C-4 tumors treated with C002 survived longer than did mice treated with Saline or C134, while mice treated with M002 differed in survival statistically from those treated with Saline [Log Rank (Mantel-Cox) test: C002 vs Saline, p=0.0072; C002 vs C134, p=0.0108; C002 vs M002, p=0.0898; M002 vs Saline, p=0.0498]. *p<0.05, **p<0.01, ***p<0.001, ****p<0.0001. [file Image_1.jpeg]

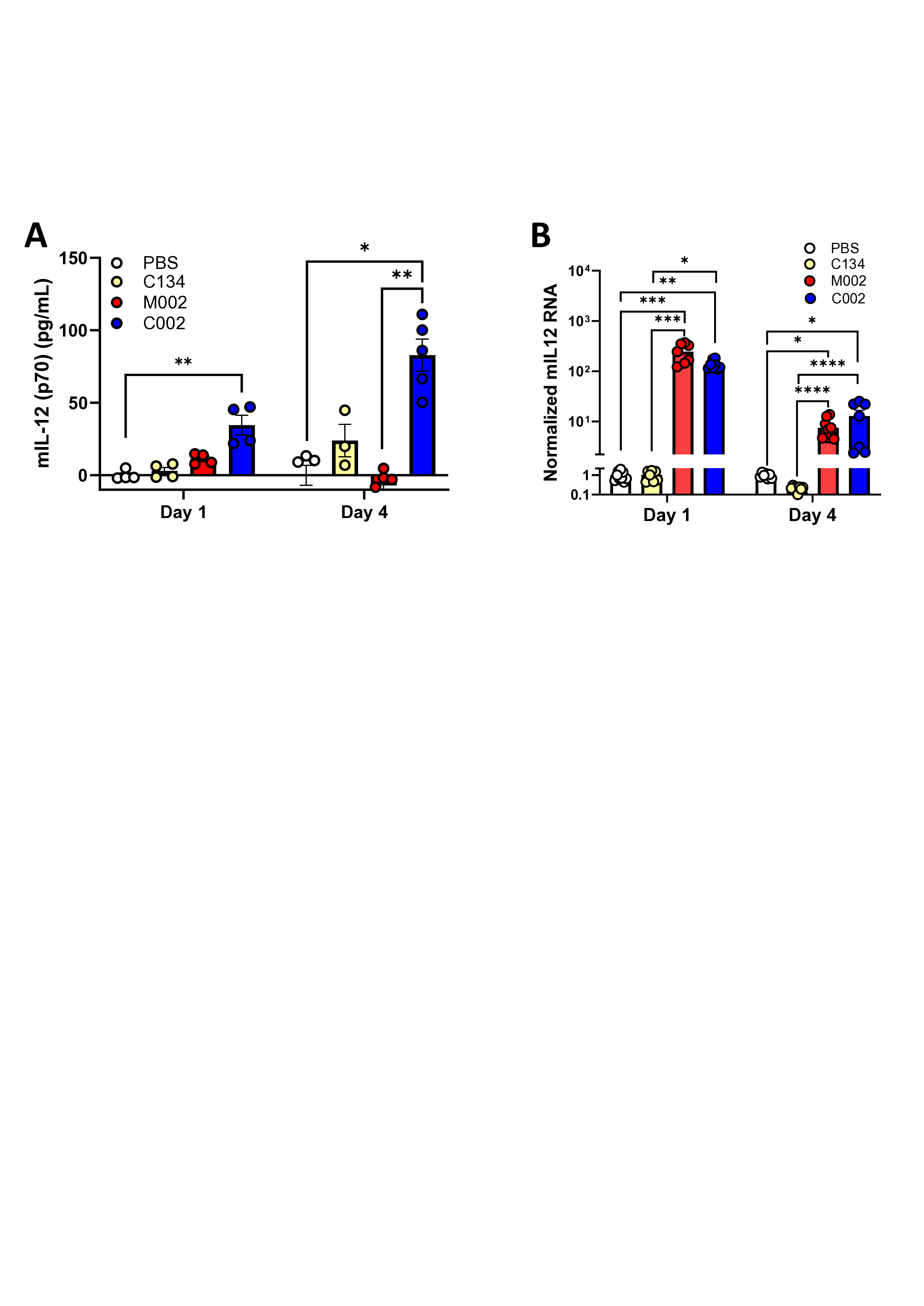

Supplement: SUPPLEMENTARY Figure 2 — IL-12 (p70) protein and transcript levels in C57BL/6-based 67C-4 MPNSTs. Three- to 4-week-old C57BL/6 mice were implanted subcutaneously with 4×10^6^ cells in 50µL of phosphate buffered saline (PBS)/flank. When tumor sizes reached 60-200mm3, animals were randomized into treatment groups by tumor size to ensure a similar average tumor size per cohort. Tumors were then treated with saline or oHSV C134, M002, or C002 (3×10^7^ PFU in 50µL PBS)/flank intratumorally (ITu). Mice were sacrificed at 1- and 4- days post-treatment, tumors harvested and homogenized by mechanical disruption. RNA was extracted from aliquots of tissue homogenates, converted to cDNA and qPCR performed to quantify IL-12 RNA levels. Another aliquot of tissue homogenates were incubated with an equal volume of SDS-free RIPA lysis buffer, centrifuged, supernatants collected, and analyzed with either mouse IL-12 (p70) ELISA MAX Deluxe Set. (A) C002 produced greater levels of IL-12 (p70) protein than all other virus cohorts produced at both 1- and 4-days following first virus treatment in 67C-4 flank tumors detected by ELISA. (B) M002-treated tumors contain abundant mIL-12 transcript levels, which are similar to or greater than C002-treated tumors at both 1- and 4-days post first virus treatment. [file Image_2.jpeg]

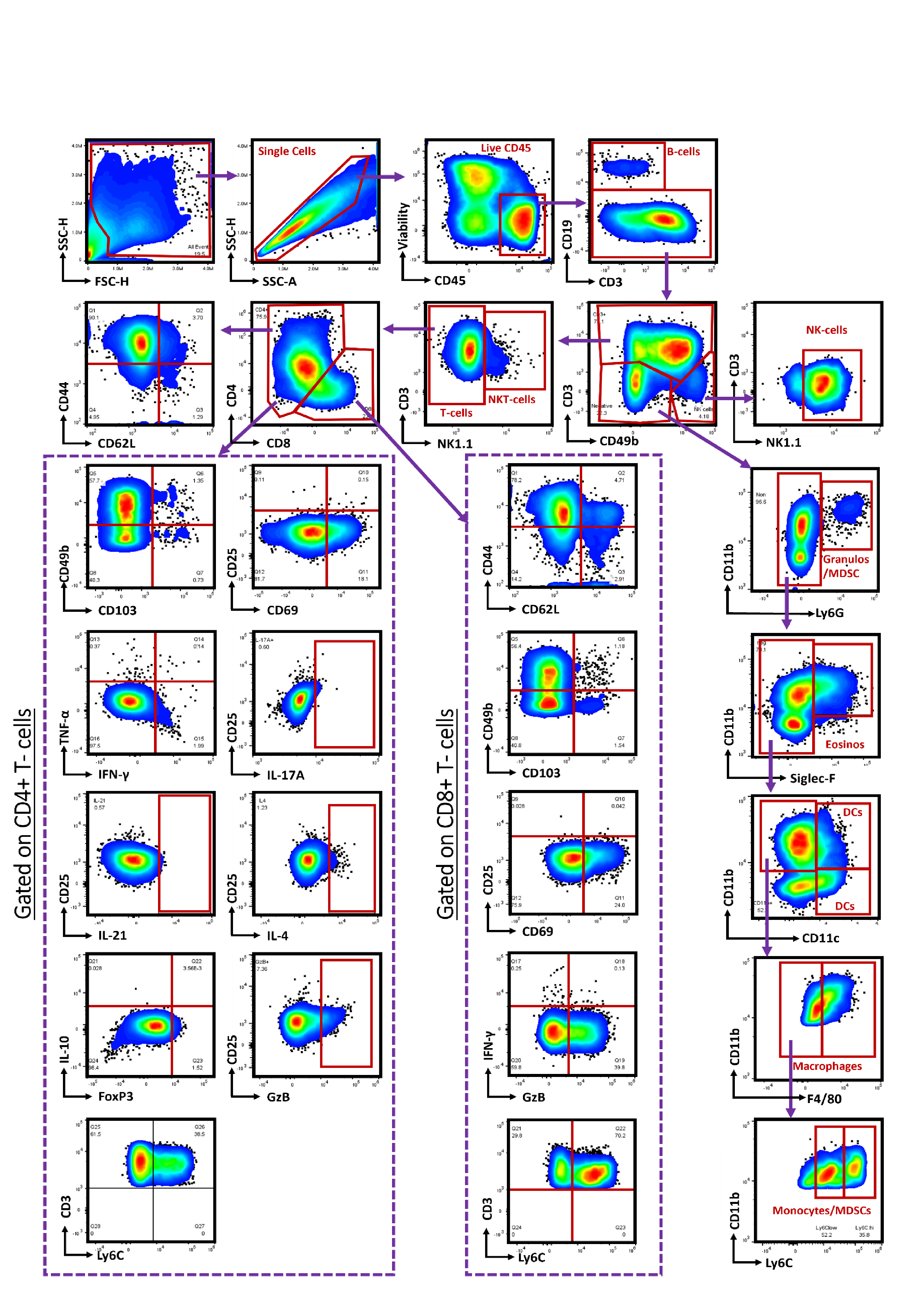

Supplement: SUPPLEMENTARY Figure 3 — Gating strategies for flow cytometric analysis of myeloid and lymphoid compositions in tumors. Tumors were treated with either PBS (n=8), C134 control (Δγ134.5, IRS1; n=8), M002 (Δγ134.5, mIL-12; n=8), or C002 (Δγ134.5, IRS1, mIL-12; n=8). On Day 11 post-treatment (Day 4, post second oHSV treatment), mice were sacrificed, and tumor infiltrates isolated. Tumor infiltrating leukocytes were stained with fluorescent labelled antibodies and analyzed using spectral flow cytometry. In the gating strategy, debris exclusion and leucocyte selection were initiated using forward scatter and side scatter area. Viable CD45(+) cells were identified through gating based on their low expression of Zombie NIR viability stain and high expression of CD45. Within the live CD45(+) gate, B cells (CD19+) were gated. Among CD19(-) population, CD3(+) cells were differentiated from CD49b(+) cells. NK cells were gated among CD3(-)CD49b(+) cells and recognized as NK1.1(+) expressing cells. Among CD3(+) cells, Natural Killer T (NKT) and T cells were differentiated based on their expression of NK1.1. T cells with CD3(+)NK1.1(-) expression were further gated for CD4(+) T helper (Th) or CD8(+) T cytotoxic (Tc) cells. CD4(+) T cells were differentiated as regulatory T cells (Tregs) based on their expression of FoxP3(±)IL-10 expression. CD4(+) or CD8(+) T cells were individually gated for their surface expression of Ly6C, CD44, CD62L, CD49b, CD103, CD25 and CD69 as well as for their intracellular cytokines (TNF-α, IFN-γ, IL-4, IL-17A, IL-21 and Granzyme B for CD4; IFN-γ and Granzyme B for CD8) expression. Combination of CD44 and CD62L expressions were used to identify naïve (CD44-CD62L+), central memory (TCM; CD44+CD62L+) and effector/ effector memory (TE/TEM; CD44+CD62L-) populations. Likewise, CD49b(±)CD103 expression was used to characterize tissue resident memory T (TRM) cells. Activation of T cells were assessed through their expression of CD25(±)CD69. Sequential gating was performed among CD19( [file Image_3.jpeg]

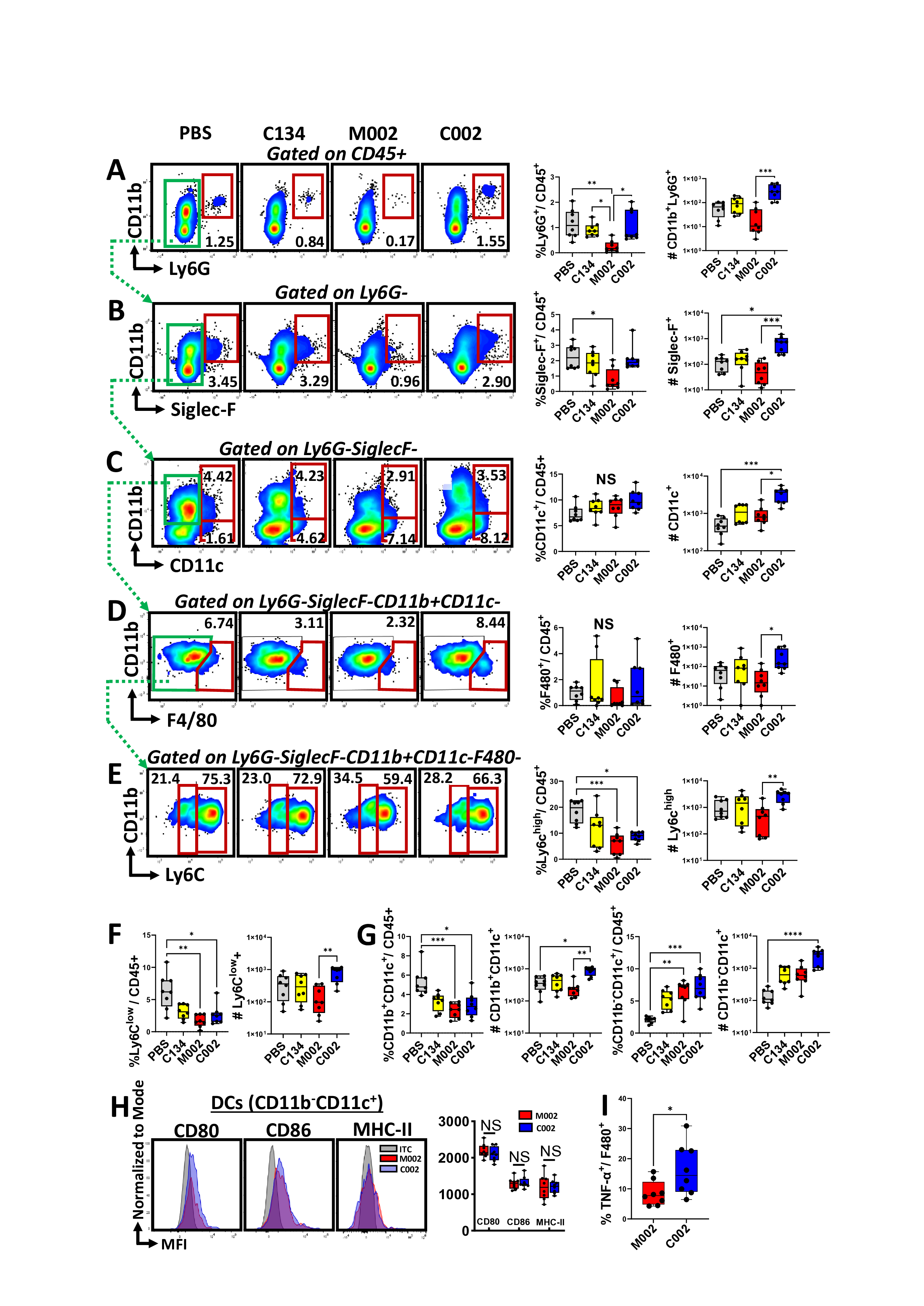

Supplement: SUPPLEMENTARY Figure 4 — Myeloid compositions of 67C-4 tumor infiltrates. Tumors were treated with either PBS (n=8), C134 control (Δγ134.5, IRS1; n=8), M002 (Δγ134.5, mIL-12; n=8), or C002 (Δγ134.5, IRS1, mIL-12; n=8). On Day 11 post-treatment (Day 4, post second oHSV treatment), mice were sacrificed, and tumor infiltrates isolated. Tumor infiltrating leukocytes were stained with fluorescent labelled antibodies and analyzed using spectral flow cytometry. Sequential (sub-) gating was performed to identify granulocytes, eosinophils, dendritic cells, macrophages and monocytes within the tumor infiltrates and the frequenceis were expressed as percentage composition of CD45(+) cells. Representative flow plots, frequenceis and absolute quantities of (A) Ly6G(+) granulocytes/granulocytic myeloid derived suppressor cells (gMDSCs), (B) Ly6G(-)Siglec-F(+) eosinophils, (C) Ly6G(-)Siglec-F(-)CD11c(+) dendritic cells, (D) Ly6G(-)Siglec-F(-)CD11b(+)CD11c(-)F4/80(+) macrophages, and (E) Ly6G(-)SiglecF(-)CD11b(+)CD11c(-)F4/80(-)Ly6CHI inflammatory monocytes among the groups. (F) Comparaision of frequency and absolute quantity of Ly6G(-)SiglecF(-)CD11b(+)CD11c(-)F4/80(-)Ly6CLO patrolling monocytes among the treatment groups. (G) Frequencies and absolute quantities of CD11b(+)CD11c(+) and CD11b(-)CD11c(+) dendritic cells. (H) Mean fluorescence intensity (MFI) of CD80, CD86 and MHC-II expression on CD11b(-)CD11c(+) dendritic cells between M002 and C002 treated tumors. (I) Differences in the TNF-α expressing CD4+ T cells between M002 and C002 treated tumors. [file Image_4.jpeg]

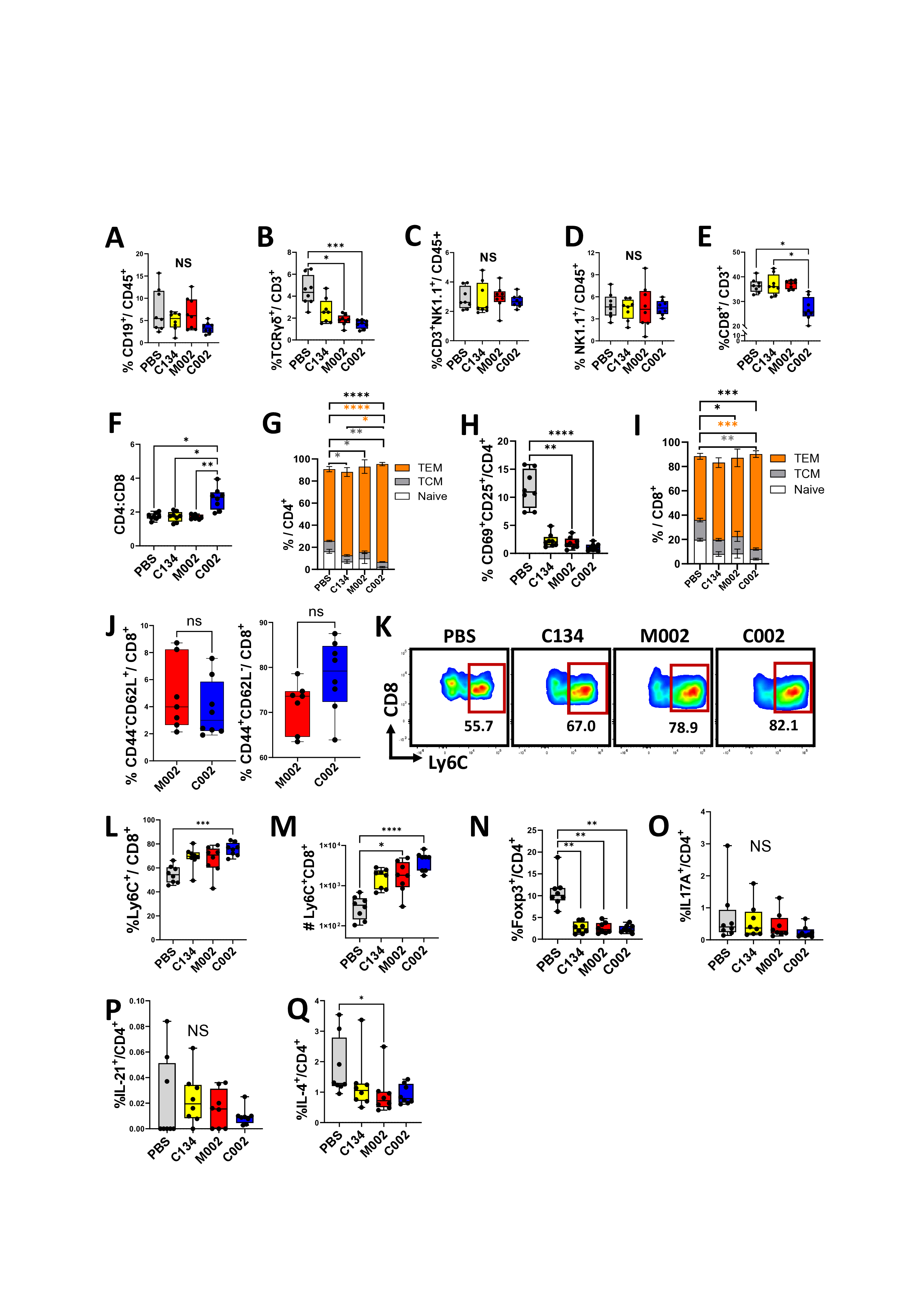

Supplement: SUPPLEMENTARY Figure 5 — Characterization of Immune infiltrates among saline and oHSV treated 67C-4 tumors. PBS (n=8) or oHSVs (C134, n=8; M002, n=8 and C002, n=8) treated tumors were harvested on Day 11 post-treatment. Tumor infiltrating leukocytes were stained with fluorescent labelled antibodies and analyzed using spectral flow cytometry. (A) Proportions of CD19(+) B cells among CD45(+) infiltrates. (B) Proportions of γδ T cells within total T cells. (C) Proportions of natural killer T (NKT) cells and (D) NK cells within CD45(+) infiltrates. (E) Proportions of CD8(+) cytotoxic T (Tc) cells among total T cells. (F) CD4:CD8 ratio within intratumoral T cells. (G) Proportions of CD44(-)CD62L(+) naïve, CD44(+)CD62L(+) central memory (TCM) and CD44(+)CD62L(-) effector/ effector memory (TE/TEM) CD4(+) T cells. (H) Frequencies of CD69(+)CD25(+) activated CD4(+) T cells. (I) Proportions of naïve, central memory (TCM) and effector/ effector memory (TE/TEM) CD8(+) T cells. (J) Frequencies of naïve and TE/TEM CD8(+) T cells between C002 and M002 treated tumors. (K) Representative flow plot showing differences in the Ly6C expression on CD8(+) T cells among the treatement groups. (L) Frequencies and absolute quantities (M) of Ly6C(+)CD8(+) T cells. (N) Frequenceis of CD4+FoxP3+ regulatory T cells (Tregs). (O) Frequenceis of IL-17A expressing T helper 17 (Th17) cells and (P) IL-21 expressing T- follicular helper (Tfh), and (Q) IL-4 expressing Th2 cells within CD4(+) cells. Results are presented as box and whisker plots showing the median, with 25–75 percentile range as the box and 5–95 percentiles as the whiskers. Differences in the frequencies and absolute quantities among the groups were compared using Kruskal-Wallis test with Dunn’s post-hoc analysis for multiple comparison. Mann-Whitney test was performed to assess the differences in the frequencies between two groups (J). *p<0.05, **p<0.01, ***p<0.001, ****p<0.0001. [file Image_5.jpeg]

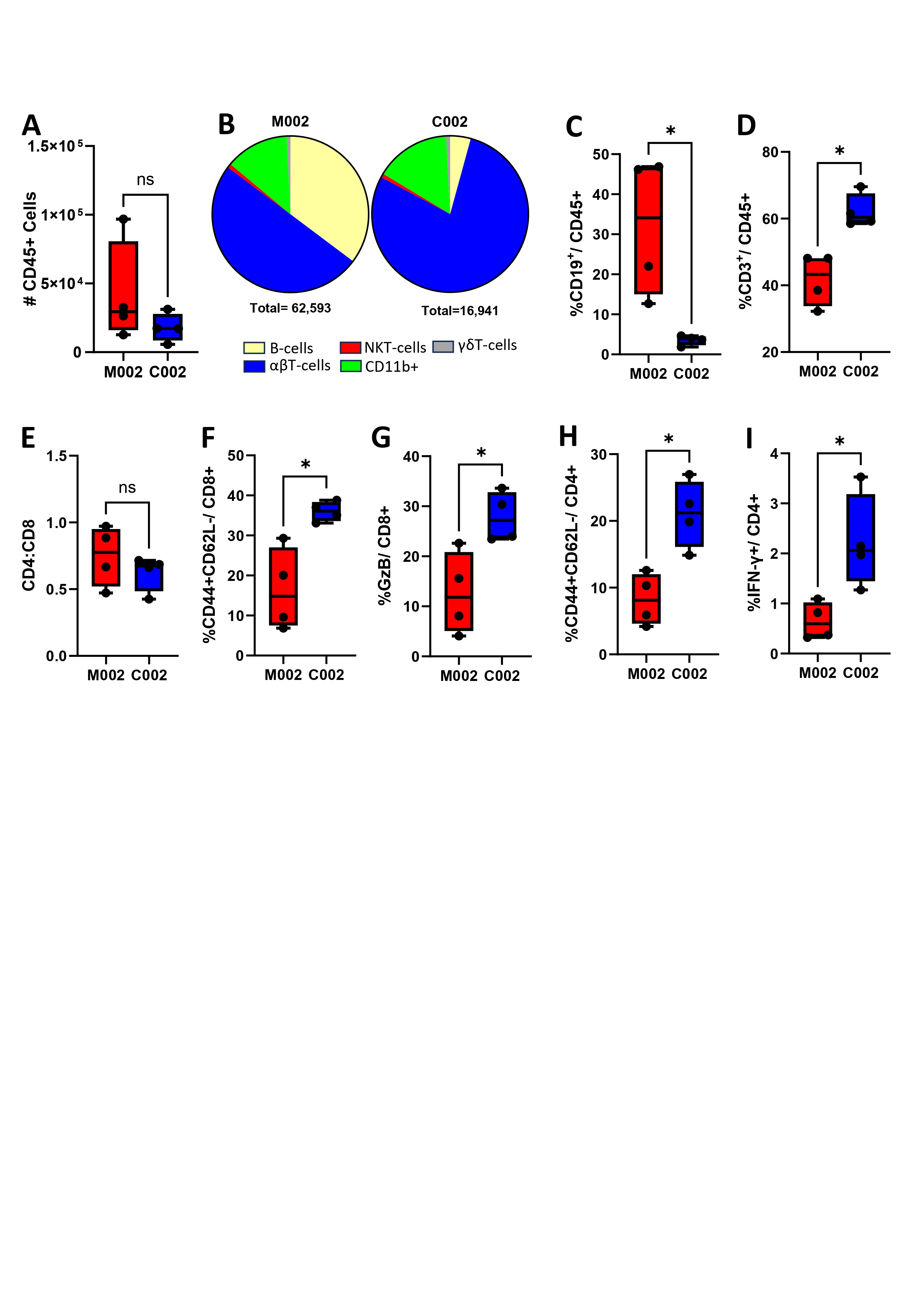

Supplement: SUPPLEMENTARY Figure 6 — Characterization of Immune infiltrates among saline and oHSV treated B109 tumors. B109 tumors were treated with either M002 (n=4), or C002 (n=4). On Day 5 post-treatment, mice were sacrificed, and tumor infiltrates isolated. Tumor infiltrating leukocytes were stained with fluorescent labelled antibodies and analyzed using spectral flow cytometry. (A) Absolute numbers of tumor-infiltrating CD45(+) immune cells between the treatment groups. (B) Pie-chart showing the percentage composition of immune cells types within the treated tumors. (C) Frequenceis of intratumoral CD3(-)CD19(+) B cells and (D) CD3(+)CD19(-) T cells between the groups. (E) Ratio of T-helper and T-cytotoxic (CD4:CD8). (F) Proportion of CD44(+)CD62L(-) effector/effector memory CD8(+) T cells. (G) Frequencies of Granzyme-B (GzB) expressing CD8(+) T cells between the treatment groups. (H) Proportion of CD44(+)CD62L(-) effector/effector memory CD4(+) T cells. (I) Frequencies of Interferon-γ (IFN-γ) expressing CD4(+) T cells between the treatment groups. Differences in the frequencies and absolute quantities between the M002- and C002- treated tumors were compared using Mann-Whitney upaired t-test. *p<0.05, **p<0.01, ***p<0.001, ****p<0.0001. [file Image_6.jpeg]
